# Urban Tree Growth and Drought Responses Show Evidence of Climate Resilience

**DOI:** 10.1111/gcb.70281

**Published:** 2025-06-09

**Authors:** Manuel Esperon‐Rodriguez, Matthew Brookhouse, Sally A. Power, Diego Avi, Thomas Baer, Paul D. Rymer, Mark G. Tjoelker

**Affiliations:** ^1^ Hawkesbury Institute for the Environment, Western Sydney University Penrith New South Wales Australia; ^2^ School of Science Western Sydney University Penrith New South Wales Australia; ^3^ Fenner School of Environment and Society Australian National University Acton Australia; ^4^ Forest Research, Forest Resources and Product Development Group Northern Research Station Midlothian UK

**Keywords:** cities, dendroecology, drought, heatwaves, multi‐year drought, tree rings, urban forests

## Abstract

Climate change has a negative impact on the vitality of forests, and drought and heatwaves are the most influential abiotic stressors that contribute to tree health decline and mortality. Urban trees are not only vulnerable to climate change, but they also face harsh environmental conditions, including the urban heat island effect, limited soil volume and water availability. Therefore, the long‐term sustainability of urban forests relies on healthy and thriving trees and the identification of species that are resilient to climate change. Thus, it is fundamental to understand how urban trees respond to environmental conditions, including climate. This study investigates how urban trees respond to both long‐term climatic conditions and episodic extreme climate events. We evaluated variation in urban tree growth across differing climates by reconstructing growth histories and developing drought response indices. We selected 10 tree species planted in seven cities distributed along temperature and precipitation gradients across the Australian continent. We determined spatial and temporal patterns of tree‐ring growth in relation to extreme climate events. We found significant differences among cities, suggesting that local environmental conditions significantly influence tree growth. While some species showed fast annual growth in cool and wet cities, other species had similar growth across all cities or even faster growth in hot and dry cities. Urban trees generally responded positively to wetter conditions during the warmest month, which might be related to longer growing seasons and water availability. We found a positive effect of extreme hot conditions on growth, suggesting that urban trees might be well adapted to warm urban environments. Species climate‐growth relationships can help guide species selection to maximize benefits delivered by urban forests and minimize environmental and socio‐economic losses under current and future climates.

## Graphical abstract

Urban trees face increasing risks from climate change, including drought and heatwaves, which threaten their growth and survival. By studying 10 tree species across seven Australian cities, we found that local climate strongly influences tree growth, and that some species are well adapted to urban climates. Our findings can help guide the selection of resilient species to ensure healthy urban forests in a changing climate.

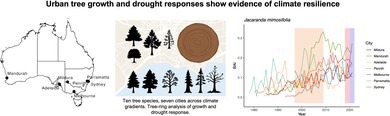

## RESUMEN

El cambio climático tiene un impacto negativo en la vitalidad de los bosques, y la sequía y las olas de calor son los factores abióticos que contribuyen más al deterioro de la salud y la mortalidad de los árboles. Los árboles urbanos no solo son vulnerables al cambio climático, sino que también enfrentan condiciones ambientales adversas, como el efecto de isla de calor urbana y la disponibilidad limitada de suelo y agua. Por lo tanto, la sostenibilidad a largo plazo de los bosques urbanos depende de la salud y el vigor de los árboles, así como de la identificación de especies resilientes al cambio climático. Así, es fundamental comprender cómo responden los árboles urbanos a las condiciones ambientales, incluido el clima. Este estudio investiga cómo los árboles urbanos responden tanto a condiciones climáticas a largo plazo como a eventos climáticos extremos episódicos. Evaluamos la variación en el crecimiento de árboles urbanos en diferentes climas mediante la reconstrucción de historias de crecimiento y el desarrollo de índices de respuesta a la sequía. Seleccionamos 10 especies de árboles plantadas en siete ciudades distribuidas a lo largo de gradientes de temperatura y precipitación en el continente australiano. Determinamos los patrones espaciales y temporales del crecimiento de los anillos de los árboles en relación con eventos climáticos extremos. Encontramos diferencias significativas entre ciudades, lo que sugiere que las condiciones ambientales locales influyen de manera importante en el crecimiento de los árboles. Mientras que algunas especies mostraron un rápido crecimiento anual en ciudades frías y húmedas, otras especies tuvieron un crecimiento similar en todas las ciudades o incluso un crecimiento más rápido en ciudades cálidas y secas. En general, los árboles urbanos respondieron positivamente a condiciones más húmedas durante el mes más cálido, lo que podría estar relacionado con temporadas de crecimiento más largas y una mayor disponibilidad de agua. Encontramos un efecto positivo de las condiciones extremadamente cálidas sobre el crecimiento, lo que sugiere que los árboles urbanos podrían estar bien adaptados a ambientes urbanos cálidos. Las relaciones entre el clima y el crecimiento de las especies pueden ayudar a guiar la selección de especies para maximizar los beneficios de los bosques urbanos y minimizar las pérdidas ambientales y socioeconómicas bajo los climas actuales y futuros.

## 1 Introduction

Climate change and rising global temperatures have a negative impact on the vitality of forests and increase the risk of tree health decline and mortality (e.g., Anderegg et al. 2013; Klockow et al. 2018). These impacts are becoming more evident with the increasing frequency and severity of extreme weather events, including the duration and intensity of heatwaves. Additionally, recent changes in atmospheric circulation patterns can lead to shifts in rainfall seasonality, resulting in more frequent and severe drought conditions (Duffy et al. 2015; IPCC 2023; Lemus‐Canovas et al. 2024; Perkins‐Kirkpatrick and Gibson 2017; Schiermeier 2018). Together, drought and heatwaves are regarded as the most influential abiotic stressors contributing to tree mortality in the context of climate change (Allen et al. 2015; Bradford et al. 2020).

In addition to the impacts on natural landscapes, climate change also threatens the health and persistence of urban forests (Esperon‐Rodriguez et al. 2022). Urban forests—i.e., all trees and shrubs in a city, present in streets, parks, woodlands, abandoned sites, and residential areas (Miller et al. 2015)—also face harsh environmental conditions, particularly those planted in streetscapes (i.e., areas along streets, including sidewalks, medians, and roadside verges). These conditions include multi‐year droughts (Moser et al. 2018; Rötzer et al. 2021), increased risk of heatwaves associated with the urban heat island effect (Ferguson and Woodbury 2007), limited soil and water, and even vandalism (Esperon‐Rodriguez et al. 2023; Nitschke et al. 2017; Richardson and Shackleton 2014), all of which can reduce tree vitality and increase the risk of dieback and mortality. Furthermore, urban forests often comprise non‐native species that have been introduced to cities for various aesthetic, functional, or ecological purposes (Gaertner et al. 2017). Some exotic species can be chosen for their broad tolerance to urban conditions (Sjöman et al. 2016; Zerga et al. 2021). However, some exotic species may be subjected to environments that differ substantially from their native habitats, potentially exceeding their evolved physiological tolerances (Esperon‐Rodriguez et al. 2022; Kendal et al. 2018). This mismatch between species‐specific adaptations and urban environmental pressures can lead to increased stress and reduced performance of urban trees (Esperon‐Rodriguez, Gallagher, et al. 2025). These conditions can compromise the delivery of socio‐economic and environmental services provided by urban forests, notably heat mitigation, biodiversity conservation, and improvement of human health and well‐being (Escobedo et al. 2019; Lin et al. 2019).

The long‐term livability of cities relies on healthy and thriving urban forests. Thus, identifying and planting species that are resilient to climate change is a key element of modern urban‐forest planning (Brandt et al. 2016). One pathway to developing this knowledge is to quantify how the growth of existing urban forests is affected by climate variability. However, to date, research on this topic has been conducted mainly at the local level (e.g., Dervishi et al. 2022; Locosselli et al. 2024; Nitschke et al. 2017; Savva et al. 2006), which limits broader interpretations of species' responses to climate. To fill this gap, climate‐envelope modelling studies have quantified the vulnerability of urban trees to future climate change (e.g., Kim et al. 2020; Lin et al. 2019; Yang 2009; Zhang and Brack 2021). These studies show that many tree species are at risk under current and future urban climatic conditions based on climate tolerance modelling. A recent assessment of global climate risk reported that by 2050, more than 70% of tree and shrub species planted in 164 global cities will be at risk from projected changes in climate under a high‐emissions scenario (Esperon‐Rodriguez et al. 2022). Climate‐envelope modelling studies, however, cannot account for microhabitat (e.g., soil) and management (e.g., irrigation) conditions, nor evaluate how trees respond to different environmental conditions and adjust locally to climate. Additionally, models may identify geographic areas that are climatically suitable for survival specifically but not necessarily optimal for urban tree growth (Puchałka et al. 2024). Therefore, one must consider data on species' ecophysiology and growth, which can provide valuable information on how urban trees respond to both long‐term climate trends and short‐term meteorological extremes.

Dendrochronology is a powerful tool to determine the impact of climate on growth rates of trees and their change through time and can be used to evaluate relationships between climate and tree growth (e.g., Brookhouse et al. 2008, D'Orangeville et al. 2018, Fritts 2001, Helama et al. 2012, Nitschke et al. 2017, Rozas et al. 2009). Although implementation in urban forests has been limited to date, several dendrochronological studies have proved its value to evaluate urban tree growth (e.g., Bartens et al. 2012; Catton et al. 2007; Gillner et al. 2014; Helama et al. 2009; Nitschke et al. 2017).

This study investigates how urban trees respond to both long‐term climatic conditions and episodic extreme climate events, which together represent critical environmental factors influencing tree growth in urban environments. We evaluated relationships between urban tree growth and climate by reconstructing growth histories and estimating drought response indices of 10 tree species commonly planted in seven cities distributed along temperature and precipitation climatic gradients across the Australian continent. Our study was guided by several hypotheses: (1) Urban tree growth will exhibit significant variation among species and cities, with climate variables playing a key role in explaining growth patterns across both taxonomic and geographic contexts; (2) extreme climate events, specifically drought and high temperatures, will have measurable effects on the radial growth of urban trees, with the nature and magnitude of these effects varying among species; (3) urban trees may exhibit greater tolerance or reduced sensitivity to extreme heat—compared to non‐urban trees—due to adaptation to warmer microclimates and extended growing seasons in urban environments; and (4) species‐specific climate‐growth relationships will be identifiable. To test these hypotheses, we examined the role of climate extremes (i.e., drought and high temperature) on radial growth by quantifying climate–radial growth relationships. Our findings aim to provide insights for developing guidelines for climate‐resilient urban tree selection.

## 2 Materials and Methods

### 2.1 Australian Cities and Tree Species

Seven cities across the Australian continent were selected based on the availability of urban tree inventories and their respective climates. Specifically, climatic stratification aimed to maximize gradients in both temperature and precipitation. The selected cities included Adelaide (South Australia), Mandurah (Western Australia), Melbourne (Victoria), Mildura (Victoria), Parramatta (New South Wales, NSW), Penrith (NSW), and Sydney (NSW). The coolest and warmest cities in terms of mean annual temperature (MAT) were Melbourne and Penrith, respectively, while the wettest and driest cities were Sydney and Mildura, respectively (Table 1). These cities have limited management interventions after the tree establishment period (> 2 years).

**TABLE 1:** Mean annual temperature (MAT, °C), maximum temperature of the warmest month (MTWM, °C), annual precipitation (AP, mm), precipitation of the driest quarter (PDQ, mm), summer precipitation (SP, mm; December, January, February), de Martonne Aridity Index (*I*_DM_), and Pinna Combinative Index (*I*_P_) of seven Australian cities.

City	MAT	MTWM	AP	PDQ	SP	*I*_DM_	*I*_P_	Species
Sydney	22.9	28	1259	182	315	9.6	7.1	AcNe, CeAu,GlTr, JaMi, LiSt, MaGr, PlAc, PyCa, RoPs, UlPa
Parramatta	23.7	29.8	974	126	304	5.3	4.3	AcNe, CeAu,GlTr, JaMi, LiSt, MaGr, PlAc, PyCa, RoPs, UlPa
Penrith	23.9	30.3	790	92	283	3.7	3	AcNe, CeAu,GlTr, JaMi, LiSt, MaGr, PlAc, PyCa, RoPs, UlPa
Mandurah	25.3	33.2	676	33	40	18.9	9.5	AcNe, GlTr, JaMi, LiSt, MaGr, PlAc, PyCa, RoPs, UlPa
Melbourne	20.3	27.9	651	105	154	21.7	13.5	GlTr, JaMi, LiSt, MaGr, PlAc, PyCa, RoPs, UlPa
Adelaide	21.9	29.5	473	50	63	15	8.1	CeAu, GlTr JaMi, MaGr, PlAc, PyCa, RoPs, UlPa
Mildura	24.6	33.8	348	39	97	1.7	1	GlTr, JaMi, LiSt, PlAc, PyCa, UlPa

*Note:* Species indicates the urban tree species sampled in each city in this study. Cities are ordered from wettest to driest based on their annual precipitation. Species abbreviations are as follows: AcNe = *Acer negundo* ; CeAu = *Celtis australis* ; GlTr = *Gleditsia triacanthos* ; JaMi = *Jacaranda mimosifolia* ; LiSt = *Liquidambar styraciflua* ; MaGr = *Magnolia grandiflora* ; PlAc = *Platanus acerifolia* ; PyCa = *Pyrus calleryana* ; RoPs = *Robinia pseudoacia*; and UlPa = *Ulmus parvifolia* .

We selected a set of 10 tree species planted across the seven cities; nine of these were deciduous and one evergreen ( *Magnolia grandiflora* ). These species were selected based on their presence and abundance in streetscapes across the seven cities. All species are exotic to Australia and known to produce distinct annual growth rings. This focus on non‐native species was necessary because most Australian native trees generally lack discernible annual growth rings. The absence of clear annual rings in native species makes them unsuitable for dendrochronological analyses, which rely on well‐defined yearly growth patterns. In contrast, the selected exotic species, commonly used in Australian urban forestry (Esperon‐Rodriguez et al. 2019), provided reliable annual ring formation, allowing for accurate assessment of growth patterns and climate responses over time. Additionally, these species have different climates of origin based on their known global distributions and were pre‐identified as vulnerable or resilient to climate based on previous bioclimatic modeling of climate risk (Esperon‐Rodriguez et al. 2022, 2019) (Table S1). Using urban tree inventories, we located individual trees of each species for sampling in each city. The final species selected included *Acer negundo, Celtis australis, Gleditsia triacanthos, Jacaranda mimosifolia, Liquidambar styraciflua*, *M. grandiflora* , *Platanus acerifolia, Pyrus calleryana, Robinia pseudoacacia*, and *Ulmus parvifolia* (Table 2).

**TABLE 2:** Type of growth and wood of 10 urban tree species from nine families selected for sampling across seven Australian cities.

Species	Common name	Family	Native origin	Type growth	Wood	MTWM	AP	AP 5th
*Acer negundo* L.	Box elder	Sapindaceae	North America	Fast	Diffuse‐porous	19.1	1037	756
*Celtis australis* L.	European nettle tree	Cannabaceae	Southern Europe, North Africa, and Asia Minor	Fast	Ring‐porous	17.9	824	635
*Gleditsia triacanthos* L.	Honey locust	Fabaceae	Central North America	Fast	Semi ring‐porous	19.7	545	811
*Jacaranda mimosifolia* D. Don	Jacaranda	Bignoniaceae	Brazil, Argentina, and Bolivia	Fast	Diffuse‐porous	19.0	950	881
*Liquidambar styraciflua* L.	Sweetgum	Altingiaceae	North America	Medium to fast	Diffuse‐porous	20.5	895	983
*Magnolia grandiflora* L.	Southern magnolia	Magnoliaceae	Southeastern US	Slow to moderate	Diffuse‐porous	21.5	1171	1060
*Platanus acerifolia* (Aiton) Willd	London planetree	Platanaceae	Eurasia	Fast	Diffuse‐porous	17.8	1270	961
*Pyrus calleryana* Decne.	Callery pear	Rosaceae	China and Taiwan	Fast	Diffuse‐porous	19.5	741	954
*Robinia pseudoacacia* L.	Black locust	Fabaceae	Eastern US	Fast	Ring‐porous	15.2	1059	783
*Ulmus parvifolia* Jacq.	Chinese elm	Ulmaceae	Eastern Asia	Medium to fast	Ring‐porous	20.8	815	836

*Note:* Climate data, based on the species' known distribution using occurrence records from both native and introduced distributions, show mean maximum temperature of the warmest month (MTWM, °C), mean annual precipitation (AP, mm), and the 5th percentile of AP (AP 5th, mm) as a proxy for drought tolerance. Climate data were obtained from Esperon‐Rodriguez et al. (2022).

**FIGURE 1:**
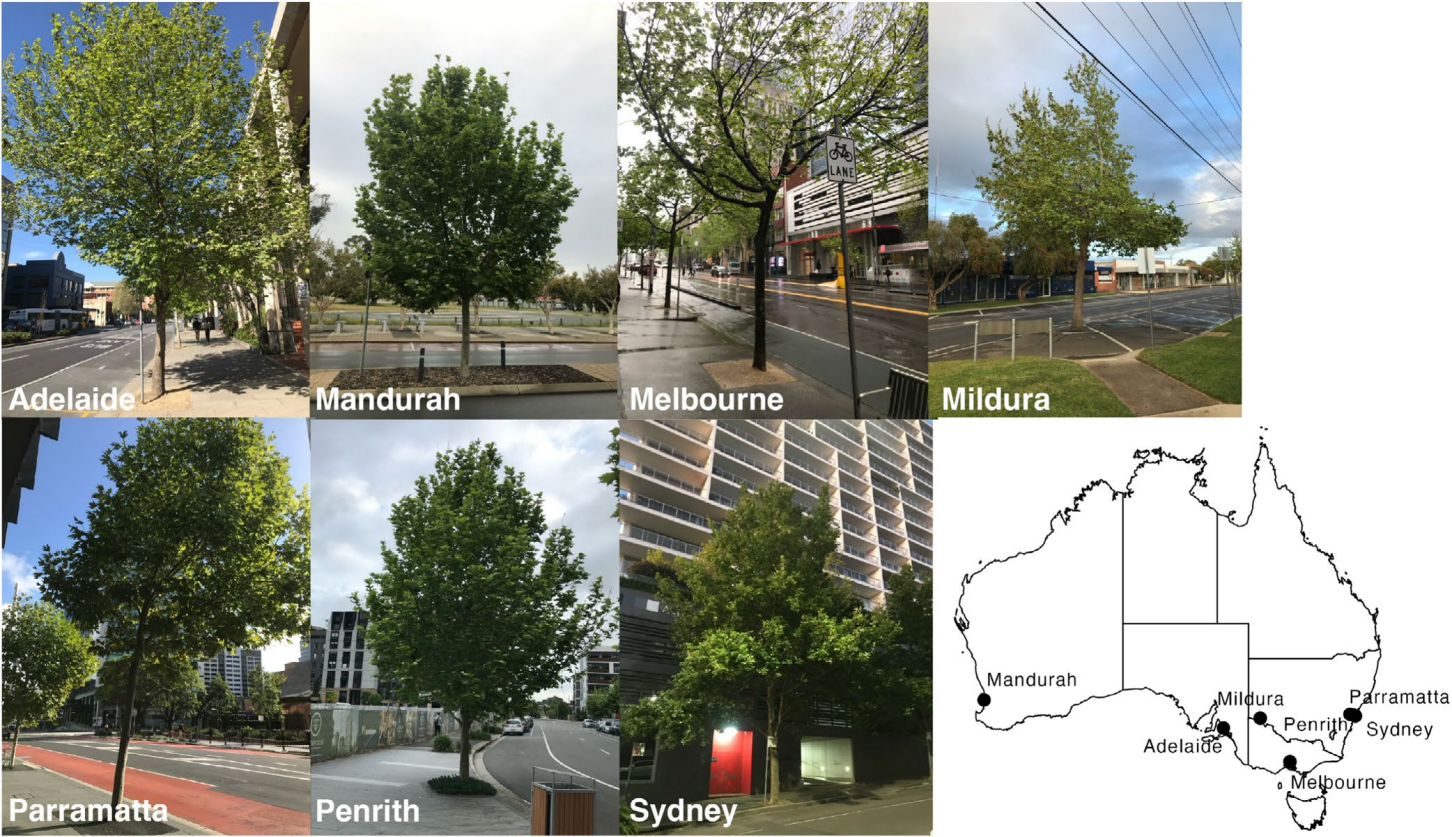
Street planting contexts and city locations for *Platanus acerifolia* in seven Australian cities. Representative street tree planting contexts are shown, along with the locations of the seven cities across the continent. Photos by M.E.‐R.

Candidate sampling locations were identified geographically using urban tree inventories provided by each city. Only public street trees were selected for sampling to standardize the general planting context (i.e., trees planted in parks or other locations were excluded). During the austral spring of 2022 (October–November), we sampled individual trees within all seven cities. We note that standardizing chosen trees by specific location or condition was not feasible given the limited number of repeated trees for each species in each city. The sampled trees encompassed a range of standard street conditions in both downtown and suburban areas. We selected trees with no apparent interference from above‐ground or below‐ground infrastructures, such as power lines. Most sampled trees were located in sidewalk pits (i.e., depressions in sidewalks designed to house the root systems of street trees) and had varying distances to buildings of different heights, particularly in downtown areas (Figure 1). We acknowledge that this variability in local growing conditions is a limitation of our study, given the complexity and heterogeneity of urban areas. However, it is an inherent challenge when conducting a multi‐city, multi‐species urban study. While we cannot completely control for these variables, our approach provides a realistic representation of the diverse conditions experienced by urban trees across different Australian cities.

Where possible, 10 healthy individuals of each species were sampled in each city. For each tree, the stem circumference was measured at the same height where cores were obtained (~1.0 m) using a measuring tape. Diameter over bark (DOB) and stem radius were calculated from the circumference data. Only trees free from any visible biotic and abiotic damage were sampled. Increment cores were removed from the main trunk (bole) of each sample tree at ~1.0 m from the ground using a 5‐mm Haglof increment borer to capture as many growth rings as possible without including distortion in rings related to any stem buttressing. Where possible, cores were removed from the northern bole aspect; when this was not possible, we collected samples from the eastern or western aspect. All cores either included the pith or rings close to the pith, providing the longest ring‐width series possible for each tree. Permission for sample collection was obtained from urban tree managers within each city.

Trees with stem diameters between 9 and 45 cm were selected for sampling to ensure a representative and consistent dataset. This size range balanced the need for sufficient annual rings to provide a meaningful chronology with the practical limitations of sampling equipment for larger trees. Trees within this diameter range likely represent a significant portion of the established urban forest and capture the typical lifespan of urban trees, which often face more stressors and have shorter lifespans than their rural counterparts (Roman et al. 2014; Smith et al. 2019). This approach allowed us to focus on trees that are established in the urban environment but are still responsive to climatic variations, providing a robust dataset for assessing climate‐growth relationships in urban forests.

### 2.2 Tree‐Ring Chronologies

Tree cores were processed and analyzed following standard dendrochronological procedures (Stokes 1996). After mounting on wooden slides and polishing with sandpaper (60 to 1200 grit), cores were scanned at 4800 dpi resolution using an Epson Perfection 4490 flatbed scanner. Tree rings were measured to 0.001 mm accuracy using *WinDendro* 2022 (Regent Instruments, Quebec, Canada). Tree‐ring series were statistically crossdated for each city and species using *COFECHA* (Grissino‐Mayer 2001), and chronologies were analyzed with *Dendroclim2002* (Biondi and Waikul 2004) to ensure accurate dating and measurement quality.

We employed the *dplR* package (Bunn 2008; Bunn et al. 2015) in *R* to detrend individual series based on an age‐dependent function for annual basal area increment (BAI). This detrending was performed for each individual tree core across all species and cities. To further ensure the accuracy and reliability of our tree‐ring data and address potential dating errors, we applied rigorous crossdating techniques using the *dplR* package in *R*. Crossdating is a critical step in dendrochronology that ensures each ring is assigned to the correct calendar year (Fritts 2012; Speer 2010). For each species and city, we constructed a master chronology using a leave‐one‐out cross‐validation approach. This involved building a master chronology for each tree core using all other cores within that species in the same city. The individual core was then correlated against the master chronology generated without its inclusion, ensuring each series was independently validated against the ensemble. We used the ‘corr.rwl.seg’ function from the *dplR* package to calculate segment‐wise correlations between individual series and the master chronology.

To assess the strength of the common growth signal, we calculated the mean interseries correlation (r̄, or r‐bar) and the Expressed Population Signal (EPS) for each city × species combination using the *dplR* package in *R*. The average r‐bar was 0.3 (SD = 0.2), with varying degrees of common signal strength among different groups. Higher r‐bar values indicate a stronger common growth signal, suggesting that trees within a group respond similarly to environmental factors. The EPS values, which quantify how well a chronology represents the theoretical population signal (Wigley et al. 1984), had an average of 0.7 (SD = 0.2). We note that while an EPS ≥ 0.85 is often used as a benchmark in dendroclimatic studies, it is not an absolute threshold, particularly in complex urban environments. As Buras (2017) describes, EPS reflects the strength of the common signal, which may include factors unrelated to climate and should not be used in isolation to reject chronologies. Additionally, EPS does not directly determine a chronology's suitability for climate reconstruction; it provides insight into the robustness of the population signal (Buras 2017). Given the variability in signal strength across different species and cities, we emphasize caution in interpreting climate‐growth relationships, particularly for chronologies with lower EPS values. Detailed r‐bar and EPS results are provided in Figure S1.

Given the variability in r‐bar and EPS across species and cities and to ensure the accuracy of our drought response analyses, we applied an adaptive thresholding approach based on the distributions of r‐bar and EPS across all city × species combinations. Instead of applying a fixed cutoff, we calculated species‐specific thresholds using the mean minus one standard deviation (Mean—1SD) of r‐bar and EPS values within each group. This approach allowed for flexibility in accounting for natural variability in growth coherence, particularly in species with inherently weaker common signals due to genetic diversity, site heterogeneity, or mixed‐age stands. City × species combinations with r‐bar and EPS values below their respective adaptive thresholds were flagged for potential exclusion. However, upon applying this method to our dataset, all city × species combinations met or exceeded their adaptive thresholds (Figure S2). Therefore, no combinations were excluded from further analyses. This method ensured a balance between retaining meaningful climate signals and minimizing the influence of weak or noisy chronologies while maintaining the integrity of the dataset.

We developed spaghetti plots of individual tree‐ring series for each species and city to assess growth pattern variability (Figure S3), followed by standardized mean chronology development (Cook 1985). This approach ensured data reliability for climate‐growth relationship analysis while balancing climate signal retention against noisy chronology influences. Detailed r‐bar, EPS statistics, and additional spaghetti plots are provided in Figures S1–S3.

### 2.3 Tree Growth Rate

We reconstructed individual tree growth using the basal area (BA) and its change through time to calculate growth rates of individual trees. Annual basal area increment per tree (BAI) was calculated as a non‐linear function of the cumulative tree radius (*R*, mm) and tree‐ring width (mm). We calculated BAI (i.e., annual growth) for each tree ring and for every tree according to Visser et al. (2023) using the following equation:

(1) $${\mathrm{BAI}}_{t}={\mathrm{BA}}_{t}-{\mathrm{BA}}_{t-1}=\pi \times \left(R_{t}^{2}-R_{t-1}^{2}\right)$$

where, *R*_*t*_ denotes the radius measured from the pith to the ring boundary having age *t*, and calculated by summing the ring widths *R*W_1_, …, *R*W_*t*_.

### 2.4 Climate Data

We obtained historic climate data from the Australian Bureau of Meteorology (<www.bom.gov.au>) using meteorological stations representative of each city and sampling site for the period 1950–2022 (Table S2). Stations were selected based on their proximity to the sampled sites and their data completeness. We used the monthly precipitation and temperatures from historical observations and determined annual precipitation (AP), precipitation of the driest month (PDM), precipitation of the driest quarter (PDQ), summer precipitation (SP; Austral summer: December, January, February), precipitation of the wettest month (PWM), mean annual temperature (MAT), maximum temperature of the warmest month (MTWM), and minimum temperature of the coldest month (MTCM). While numerous climate indices could be derived, we selected these given their known relevance to tree growth and their ability to capture key aspects of the Australian climate (Esperon‐Rodriguez et al. 2019; Moles et al. 2014; O'Donnell and Ignizio 2012).

Using monthly climate records, we calculated the de Martonne Aridity Index (*I*_DM_) and the Pinna Combinative Index (*I*_P_). The *I*_DM_ is particularly relevant in urban environments where water availability is a critical factor influencing tree growth. This index is sensitive to changes in both temperature and precipitation, making it suitable for capturing the effects of climate variability on urban tree growth. This is important in urban areas where microclimates can significantly differ from surrounding regions. The *I*_DM_ has been extensively validated and used in various ecological and climatological studies (e.g., Botzan et al. 1998; Jafarpour et al. 2023; Pellicone et al. 2019), providing a robust framework for assessing the impact of climatic conditions on vegetation. The *I*_P_ combines multiple climatic factors, offering a more comprehensive assessment of climate conditions (Deniz et al. 2011; Nistor 2016). This is beneficial for understanding complex interactions between climate and urban tree growth. This index is particularly useful in urban settings where multiple climatic factors, such as temperature fluctuations and humidity levels, interact to influence tree physiology and growth. The *I*_DM_, developed by de Martonne (1925) was calculated by the following equation:

(2) $$I_{\mathrm{DM}}=\frac{P}{T+10}$$

where *I*_DM_ is the de Martonne aridity index, *P* is the annual mean precipitation in mm, and *T* is the annual mean air temperature in °C. The climatic classification based on the *I*_DM_ values is shown in Table S3.

The Pinna Combinative Index (*I*_P_), proposed by Pinna (Zambakas 1992) takes into account the precipitation and air temperature of the driest month (Deniz et al. 2011) and is given by the following relationship:

(3) $$I_{P}=\frac{1}{2}\left(\frac{P}{T+10}+\frac{12P_{d}}{T_{d}+10}\right)$$

where *P* and *T* are the annual mean values of precipitation and air temperature, respectively, and *P*_*d*_ and *T*_*d*_ are the mean values of precipitation and air temperature of the driest month, respectively. When the value of the *I*_P_ is less than 10 (*I*_P_ < 10), the climate is classified as dry, and when the value of *I*_P_ varies between 10 and 20 (10  ≤ *I*_P_ ≤ 20) the climate is characterized as semi‐dry Mediterranean (Baltas 2007).

### 2.5 Determination of Drought Response Indices and Identification of Drought Events

To represent different gradients of drought intensity and identify drought events and their effects on tree growth, we calculated the standardized precipitation evapotranspiration index (SPEI; McKee et al. 1993). The SPEI is a widely used meteorological drought index and is based on monthly precipitation time series and relates the precipitation deficit to the mean and standard deviation of the time series (Vicente‐Serrano et al. 2010). The SPEI can identify and differentiate between frequently occurring short and long drought events. The sensitivity of the SPEI to past climatic events can be modified by changing the duration of the time window used for its calculation. We used a time window of 2 and 3 months, meaning that the calculation of the SPEI value for any given month is influenced by its preceding 2 or 3 months, respectively. We chose two different time scales—two and three months—to enhance the past conditions signal and because these time scales represent biologically meaningful periods of water shortage for tree‐ring growth (George et al. 2015).

For the calculation of SPEI, we used the package *SPEI* (Beguería et al. 2017) in *R*. For each city and time scale (i.e., 2 or 3 months), we estimated: (1) the minimum SPEI in each year, indicating the severity of the drought conditions that were reached that year (severe drought: SPEI ≤ −1.50; extreme drought: SPEI ≤ −2.00; Azman et al. 2022, Tirivarombo et al. 2018); (2) the mean SPEI for the whole year; low values suggest that more months that year had low SPEI values, which could indicate that the drought‐like conditions lasted longer; and (3) the variance of the mean SPEI; a low variance indicates that drought conditions lasted longer in a particular year. In combination, these three metrics (i.e., minimum, mean, and variance) were used as an indicator for drought severity by considering both intensity and duration. We selected the years with the lowest values for all three metrics as extreme drought events. These events were then used to evaluate tree growth under drought.

For each city, we identified drought events over the period 1952 to 2022 (Table 3; Figures S4–S10). Some of these time periods coincided with El Niño and La Niña years (data from the Australian Bureau of Meteorology<http://www.bom.gov.au/climate/history/enso/>), which are opposite phases of the El Niño‐Southern Oscillation (ENSO) climate pattern. El Niño is characterized by warmer‐than‐average sea surface temperatures in the central and eastern tropical Pacific Ocean, often leading to drier conditions in eastern Australia (Chiew et al. 1998). La Niña is characterized by cooler‐than‐average sea surface temperatures in the same region, often leading to wetter conditions in eastern Australia (Huang et al. 2024). Additionally, we included the Australian Millennium Drought—an extended drought in southern mainland Australia from 1997 to 2009 (Heberger 2011), and the extreme hot and dry years from 2018 to 2020 that preceded the Black Summer, during which extensive wildfires occurred in Australia (Levin et al. 2021). These extreme conditions were often followed by subsequent La Niña years, when wet conditions predominated throughout southeastern Australia (Fasullo et al. 2023).

**TABLE 3:** Occurrence of drought events for seven Australian cities over the period 1980–2022 and corresponding tree ring time series.

City	Tree ring time series	No. events	Drought events	Event
Adelaide	1987–2022	2	2006–2008, 2014–2015	El Niño
Mandurah	1944–2022	2	2006, 2012–2013	El Niño, La Niña (2012)
Melbourne	1940–2022	2	2009, 2018–2019	La Niña (2009)
Mildura	1940–2022	2	1985, 2004	
Parramatta	1965–2022	3	1995, 2002, 2017–2018	El Niño
Penrith	1940–2022	3	1982, 1995, 2018	El Niño
Sydney	1940–2022	2	1980, 2017–2018	

*Note:* Some drought events span several years (i.e., consecutive years) and coincide with El Niño and La Niña years (Data from the Australian Bureau of Meteorology<http://www.bom.gov.au/climate/history/enso/>). See details of each city in Figures S4–S10.

To assess tree performance during drought events, we calculated three indices of drought response: (1) resistance—characterized as a tree's ability to withstand a period of high water deficit without showing a noticeable decrease in BAI (i.e., trees with a resistance > 1 do not show any decrease in annual growth rate compared to the previous years before drought); (2) recovery—the ability to increase BAI from declines experienced during a drought (i.e., recovery = 1 indicates persistence at low growth levels after a drought, whereas recovery > 1 indicates an immediate increase in growth); and (3) resilience—the capacity of a tree to recover and reach pre‐drought levels of BAI after a drought event (resilience = 1 means full restoration, resilience < 1 indicates a persistent decline in growth) (Lloret et al. 2011). These indices were calculated as follows:

(4) $$\text{Resistance}=\frac{{\mathrm{BAI}}_{\text{drought}}}{{\mathrm{BAI}}_{\mathrm{pre}-\text{drought}}}$$

(5) $$\text{Recovery}=\frac{{\mathrm{BAI}}_{\text{post}-\text{drought}}}{{\mathrm{BAI}}_{\text{drought}}}$$

(6) $$\text{Resilience}=\frac{{\mathrm{BAI}}_{\text{post}-\text{drought}}}{{\mathrm{BAI}}_{\mathrm{pre}-\text{drought}}}$$

Pre‐ and post‐drought detrended BAI were calculated as average values for a three‐year period before and after a period with drought. Resistance, recovery, and resilience indices were calculated for each individual tree based on its BAI values during specific drought events. These calculations were performed separately for each city and species, considering multiple drought events where applicable. We followed this approach to account for a delayed response to drought and cumulative effects. Further, when we compared average pre‐ and post‐drought growth between 2‐and 3‐year periods, we found no significant differences (ANOVA test, *F* = 0.03, *p* = 0.9). Growth during the drought event was calculated as the mean of detrended BAI from each drought year(s).

### 2.6 Statistical Analyses

To examine significant differences in annual radial growth (i.e., annual basal area increment, BAI) among species and cities, we used the non‐parametric Kruskal‐Wallis test. Post hoc comparisons following significant Kruskal‐Wallis tests were conducted using Dunn's test with Bonferroni correction to identify specific pairwise differences among groups. This approach ensured control over Type I error rates during multiple comparisons.

After confirming that all assumptions for parametric testing were met (normality and homogeneity of variances), we used an Analysis of Variance (ANOVA) to test differences in the three drought response indices (resistance, recovery, and resilience) among species. Tukey's Honest Significant Difference (HSD) test was applied for post hoc multiple comparisons to identify specific pairwise differences between species.

To reduce the dimensionality of the climate data and to explore the relationships among the 10 climate variables (AP, PDM, PDQ, PWM, SP, MAT, MTWM, MTCM, *I*_DM_, *I*_P_) across the sampled cities, we used Principal Component Analysis (PCA) (Jolliffe 1986). PCA is a dimension‐reduction technique that identifies orthogonal components capturing the greatest variance in the dataset (Legendre and Legendre 2012). Based on the magnitude and direction of vectors in relation to the first two principal components, which explained ~70% of the total variance, we identified three non‐correlated climate variables: AP, MAT, and *I*_P_ (Figure S11).

Following our initial PCA, we conducted a LASSO (Least Absolute Shrinkage and Selection Operator) regression analysis to complement our variable selection process. LASSO is a regularization technique that performs both variable selection and regularization, enhancing the prediction accuracy and interpretability of the resulting statistical model (Tibshirani 1996). We applied LASSO regression to our full set of climate variables, including those initially excluded after the PCA. The LASSO model was fitted using cross‐validation to determine the optimal regularization parameter (lambda). The LASSO regression identified three key variables as the most influential predictors of tree growth: PWM, *I*_P_, and MAT. The inclusion of PWM, which was not among the top variables in our PCA‐based selection, underscores the importance of considering extreme precipitation events in addition to annual averages when studying tree growth responses to climate. The confirmation of the importance of *I*_P_ and MAT by both PCA and LASSO strengthens our confidence in their relevance to tree growth patterns in our study cities.

We used generalized least squares (GLS) analysis with a Gaussian error structure to assess how detrended basal area increment (BAI) was affected by climate (PWM, *I*_P_, and MAT) and extreme climate events. GLS models with correlation = corAR1 (form = ~1|city/species) were employed to explicitly model and account for temporal autocorrelation within individual tree‐ring series, which is critical for addressing the non‐independence of observations in dendrochronological data. Detrended BAI was used to standardize growth patterns across trees of different ages and sizes and to enhance the detection of climate sensitivity in tree growth (Peters et al. 2015; Scharnweber et al. 2019; Visser et al. 2023). Critically, we used year‐specific climate data, allowing us to assess how inter‐annual variability in climate influenced tree growth. To account for potential nursery effects and transplantation stress, we removed the first 2 years of growth from all trees in our analyses. This approach allowed us to focus on growth patterns more likely influenced by urban environmental conditions.

The extreme climate events were included as a single categorical variable named “extreme climate events” to assess their impact on tree growth alongside long‐term climatic variables (PWM, *I*_P_, and MAT). This variable represented the dominant extreme climate event affecting each year, with levels including: (1) extreme heat events, specifically the Black Summer of 2019–2020; (2) La Niña years (based on the Oceanic Niño Index); and (3) the Australian Millennium Drought (1997–2009). Years not characterized by any of these extreme conditions were classified as “normal”. Years were assigned to a category based on whether those conditions were met. These events represent substantial deviations from the average climate conditions in each city. By incorporating these extreme climate events, we aimed to assess their impact on tree growth above and beyond the average climate conditions of a given city. Furthermore, the inclusion of extreme climate events ensures that both chronic (long‐term climate) and acute (episodic stressors) influences on tree growth were accounted for, providing a comprehensive understanding of how urban trees respond to environmental variability.

Models were tested using a mixed‐effects structure with both ‘city’ and ‘species’ as random intercepts to account for non‐independence of observations within cities (spatial clustering), species‐specific variation, and the nested structure where species are grouped within cities (not all species occur in all cities). City was treated as a random effect because we consider our study cities to represent a sample of potential urban environments rather than specific focal populations. This approach improves generalizability to other cities while accounting for unmeasured city‐level heterogeneity (Gelman and Hill 2007). However, we note that while including ‘city’ as a random effect account for a portion of city‐level variation, it does not fully capture all the specific factors influencing tree growth within each city. Data limitations prevented the inclusion of these factors as fixed effects in our model. Species was retained as a random effect to accommodate interspecific differences in growth patterns. Furthermore, to address temporal autocorrelation, we incorporated a correlation structure in our model using the *nlme* package (Bates et al. 2015) in *R*, which explicitly models the temporal autocorrelation. The model formula was: BAI_Detrended_ ~ PWM + *I*_P_ + MAT + extreme climate events + correlation = corAR1 (form = ~1|city/species). All variables were standardized using the ‘scale’ function in *R*, which centred each variable at its mean and scaled it to have a standard deviation of 1. Consequently, the units of the standardized variables are expressed in standard deviations from the mean, allowing for comparison across different metrics. Model performance was evaluated through the calculation of *t*‐statistic values at a significance level of *p* < 0.05. Model diagnostics included residual plots and variance inflation factors (VIF) to check for multicollinearity. All analyses were conducted using the statistical software *R* v.4.2.0 (R Core Team 2022).

## 3 Results

### 3.1 Tree Growth

We sampled 571 individual trees from all seven cities. *Magnolia grandiflora* was on average the oldest of the species sampled, while *P. calleryana* was the youngest. The oldest tree was an individual of *M. grandiflora* sampled in Sydney (*n* = 70 rings), while the youngest tree (*n* = 5 rings) was an individual of *J. mimosifolia* in Penrith (Table S4).

Across all species, average annual BAI (hereafter termed growth) differed significantly among cities (*H* = 287.2, *p* < 0.001). The fastest average growth for all species was recorded in Mildura (0.14 cm^2^ ± 0.16), the driest city in this study, whereas the slowest growth was recorded in Penrith (0.9 cm^2^ ± 0.9), the warmest city. Growth was also significantly different among species (*H* = 1229.8, *p* < 0.001), with *P. acerifolia* (0.14 cm^2^ ± 0.15) and *M. grandiflora* (0.05 cm^2^ ± 0.06) having the fastest and slowest average growth, respectively (Tables S5 and S6; Figures S12 and S13).

**FIGURE 2:**
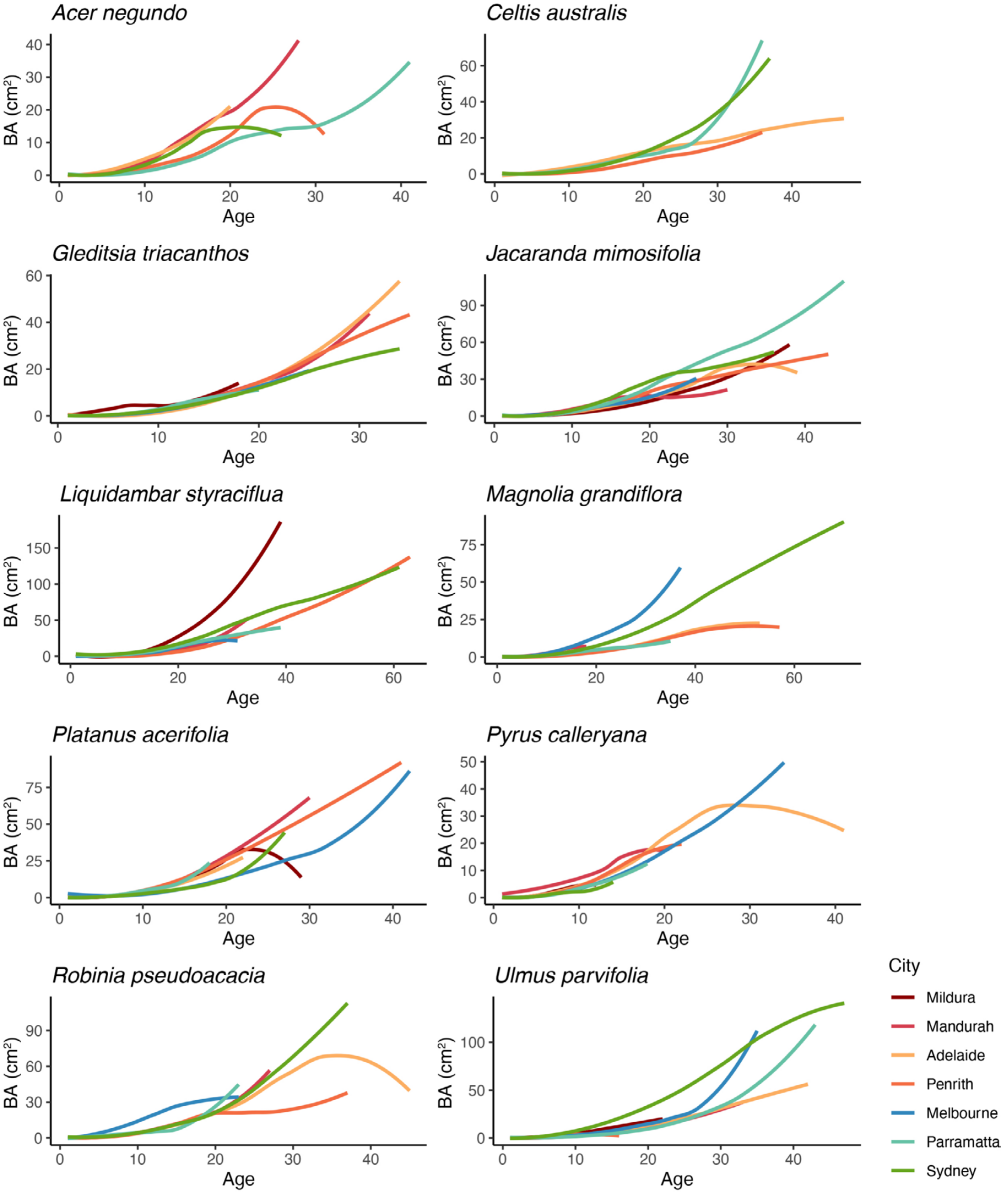
Cumulative basal area (BA) trends for 10 tree species across seven Australian cities over time. Each line represents the average growth trend for all trees in a specific city, smoothed using a generalized additive model. Note that these lines depict the average growth trajectory across different individuals, not the growth trajectory of any single tree. The apparent decrease in cumulative BA in some instances is due to variability in individual tree growth rates within the sample population, as the average is taken across trees, rather than the average of each tree. Different colours indicate different cities, illustrating potential variations in growth patterns across urban environments. Note that not all species were found in each of the seven cities. Cities are ordered from low (top, Mildura) to high (bottom, Sydney) annual precipitation. See Table S4 for the number of trees in each city.

**FIGURE 3:**
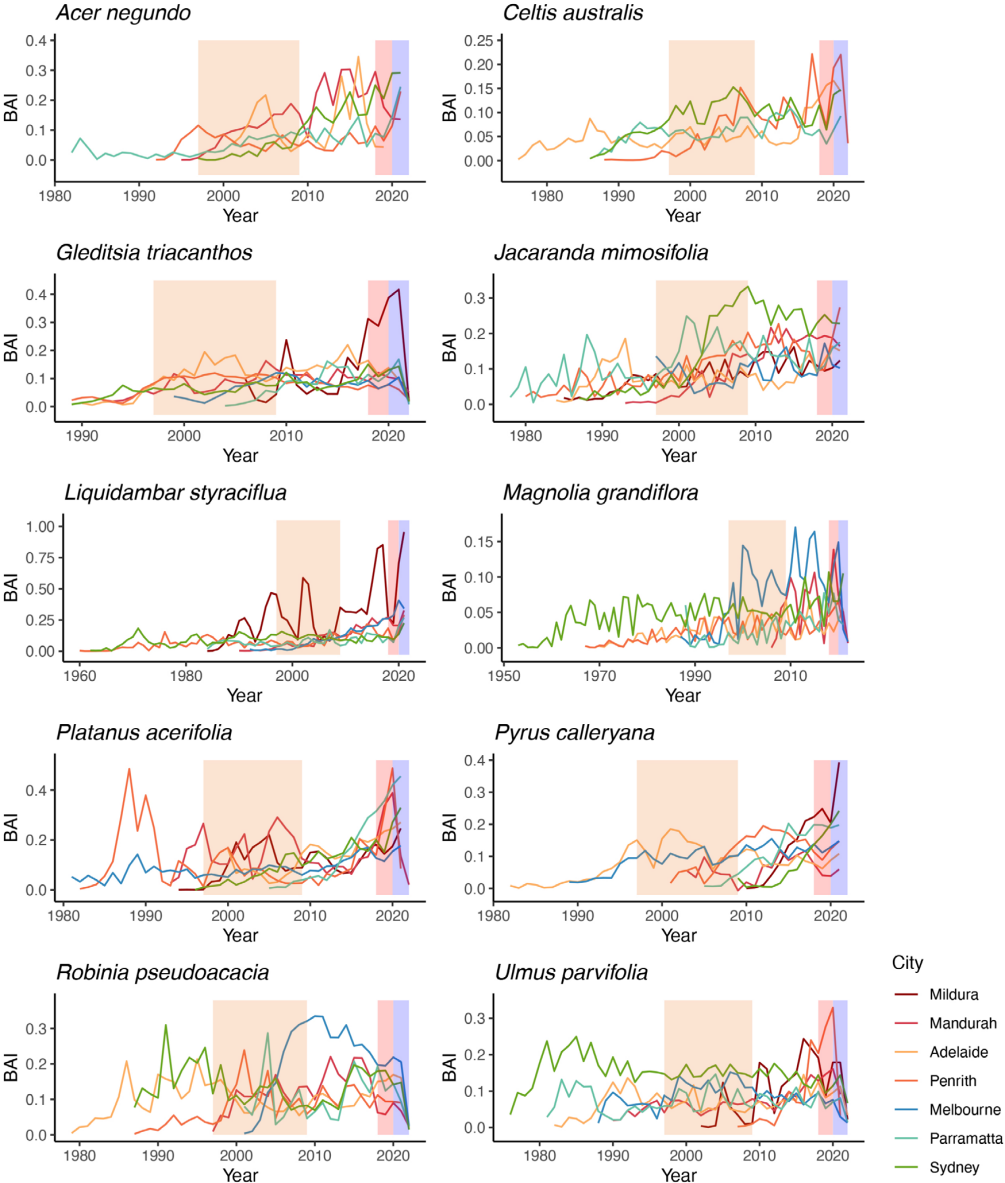
Average detrended annual basal area increment (BAI) for 10 tree species in seven Australian cities. Shaded areas indicate extreme climate events: Orange = Millennium drought; red = Black summer; blue = La Niña (cooler and wetter than average). Results represent averages across all trees planted in each city. Note that not all species were found in each of the seven cities. Detrended BAI is unitless. Cities are order from low (top, Mildura) to high (bottom, Sydney) annual precipitation. See Table S4 for the number of trees in each city.

While cumulative basal area was similar for some species in all cities where they were planted (e.g., *G. triacanthos* and *J. mimosifolia* ), other drought‐sensitive species presented great variability among cities (e.g., *A. negundo, M. grandiflora*, and *R. pseudoacacia* ) (Figure 2). A declining trend in growth over more recent years was found in some species' chronologies. This trend was particularly notable for *G. triacanthos*, for *R. pseudoacacia* , and overall, all species showed a decline in growth in the last 4 years (i.e., 2018–2022). However, we found some exceptions. *Acer negundo* growth increased in the two proximally located cities of Parramatta and Penrith during recent years (2020–2021). Similarly, *P. calleryana* had an increase in growth in all cities over this time span. Other species, such as *C. australis* , *J. mimosifolia* , and *P. acerifolia* , presented relatively consistent growth trends across all cities where they occurred (Figure 3).

### 3.2 Drought Response Indices

Across all species and cities, average drought response indices were 1.1 ± 0.4 for resistance, 1.3 ± 0.6 for recovery, and 0.2 ± 0.4 for resilience. High resistance values (i.e., > 1) indicate that trees had the ability to withstand a period of severe water deficit without showing a noticeable decrease in BAI (resistance), while high recovery values (> 1) indicate an immediate increase in growth after drought. However, some trees had a limited capacity to reach pre‐drought levels of BAI after a drought event (resilience). In general, trees were capable of resisting drought, but growth declined markedly following drought events, where trees in the drier cities—and more severe droughts—had less resilience than trees in wetter cities.

**FIGURE 4:**
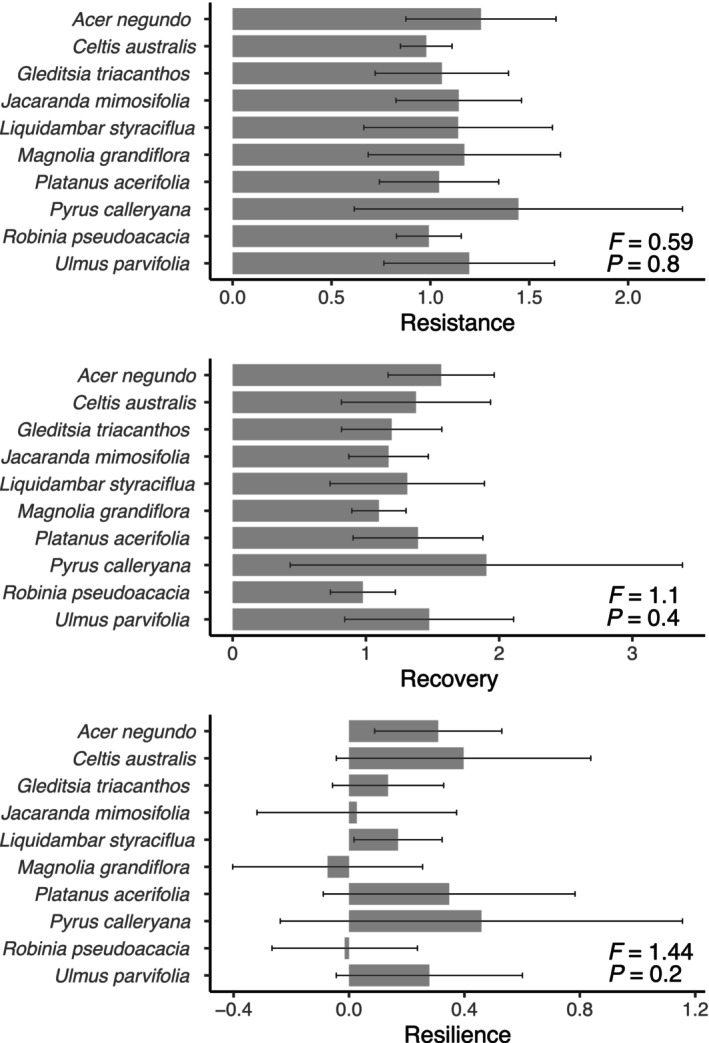
Averages and standard deviations of drought resistance, recovery, and resilience indices for 10 tree species. Indices are shown in response to two drought events across seven Australian cities (see Table 3). Indices represent species values aggregated across all cities where they are planted. Drought response indices are unitless. For differences between cities based on the Tukey's Honestly Significant Difference (HSD) test, see Table S7.

Although tree drought responses varied, we found no significant differences when we compared resistance (*F* = 0.59, *p* = 0.8), recovery (*F* = 0.4, *p* = 1.1), and resilience (*F* = 1.4, *p* = 0.2) among species (Table S7). We did not find a significant interaction effect between species and cities for any of these indices (*p* > 0.05). The highest averages of all three indices were found in Sydney for *P. calleryana* , whereas the lowest averages of both resistance and resilience were found in Mildura for *L. styraciflua* (Figure 4; Table S8). Notably, species identified as vulnerable in previous research using bioclimatic models (Table S1) did not consistently exhibit greater growth sensitivity to climate across cities or to drought events.

### 3.3 Tree Growth Model

Results from the GLS model indicated that precipitation during the warmest month (PWM) and the Pinna Combinative Index (*I*_P_) significantly influenced tree growth; increased PWM and *I*_P_ were associated with increased detrended basal area increment (BAI). Extreme climate events also significantly impacted BAI. Specifically, extreme heat events (Black Summer) and extreme wet conditions (La Niña) were associated with increased tree growth (Table 4).

**TABLE 4:** Results from the generalized least squares (GLS) model assessing the impact of climatic variables (precipitation of the warmest month, PWM; the Pinna Combinative Index, *I*_P_; mean annual temperature, MAT) and weather conditions on tree growth (detrended annual basal area increment per tree; BAI).

Response variable	Explanatory variable	Estimate	SE	*t* value	*p*
Tree growth (BAI)	(Intercept)	−0.03	**0.02**	−1.56	0.12
	PWM	0.04	**0.01**	4.25	**< 0.001**
	*I*_P_	0.02	**0.01**	2.23	**0.03**
	MAT	−0.02	**0.02**	−1.32	0.19
	Black summer	0.15	**0.03**	5.37	**< 0.001**
	La Niña	0.04	**0.02**	2.15	**0.03**
	Millennium drought	0.01	**0.03**	0.37	0.71
	Normal	0.03	**0.02**	1.84	0.07

*Note:* Weather conditions were categorized as extreme climate events (extreme heat from the Black Summer, extreme wet La Niña, and Millennium drought) or normal conditions. Weather conditions and cities were included as categorical variables, and parameter estimates are shown. Bold indicates a significant effect (*p* < 0.05).

## 4 Discussion

In this study, we sought to explore growth trends and drought responses of 10 commonly planted tree species across climate gradients in seven cities across the Australian continent. Species exhibited differing responses depending on the city in which they were planted. These differences in growth rates may reflect species' climatic and environmental preferences as well as their potential adaptive capacity. While local environmental factors, such as soil conditions or air pollution, may also influence urban tree growth, our study focused on climatic variables and extreme climate events because they represent broad‐scale drivers that are critical for understanding tree responses across multiple cities. Additionally, these factors are consistent across all sampled cities, allowing for robust comparisons.

### 4.1 Tree Growth

Our findings revealed significant variations in urban tree growth across species and cities, supporting our hypothesis that environmental conditions and species‐specific traits influence growth patterns. *Magnolia grandiflora* was the longest‐lived species, while *P. calleryana* was the youngest, highlighting differences in species longevity in urban settings. Although growth rates varied among species, most demonstrated the capacity to perform well across diverse Australian urban environments. Notably, the fastest growth occurred in Mildura, the driest city in our study, while the slowest was recorded in Penrith, which experienced some of the hottest summers across all cities, suggesting that factors beyond precipitation, such as temperature, soil conditions, or urban microclimates, may drive growth differences.

While some species (e.g., *G. triacanthos* , *J. mimosifolia* ) showed consistent cumulative basal area across cities, drought‐sensitive species (e.g., *A. negundo* , *M. grandiflora* , *R. pseudoacacia* ) exhibited greater variability. A declining growth trend over recent years was evident for most species, aligning with our hypothesis that extreme climate events—particularly prolonged drought and elevated temperatures—negatively impact urban tree growth. However, the degree of impact varied considerably among species and cities, reflecting both species‐specific sensitivities and local climate regimes. Some species demonstrated a capacity to maintain or even increase growth under warmer conditions, supporting the hypothesis that certain urban trees have adapted to, or are buffered against, urban heat. These results reinforce the need to consider both climatic and biological context when planning for urban forest resilience under climate change. Furthermore, some exceptions were observed; *A. negundo* showed increased growth in Penrith and Parramatta, while *P. calleryana* displayed a growth increase across all cities, suggesting possible adaptations to urban conditions. However, its observed faster growth rates may also reflect its younger age and partial shade tolerance, rather than innate resilience. Additionally, species like *C. australis* , *J. mimosifolia* , and *P. acerifolia* maintained relatively stable growth trends, reinforcing the idea that some tree species may exhibit resilience to climate variability. Importantly, our findings challenge the notion that selecting a few “best species” is necessary, as many species performed similarly across diverse urban environments. These findings support a broader, diversity‐focused approach to species selection (Esperon‐Rodriguez, Gallagher, et al. 2025; Paquette et al. 2021; Sjöman et al. 2016), rather than reliance on a few high‐performing taxa. Given that low species diversity is among the greatest risks facing urban forests under climate change—making them more vulnerable to pests, diseases, or extreme events—our results highlight the potential to expand species palettes without compromising urban forest performance.

Some species, however, might be more sensitive than others to climate. This is especially important for diffuse‐porous species, which are susceptible to progressive xylem cavitation—e.g., *L. styraciflua* , in response to extended growing season aridity, which in turn can have effects on tree functioning and growth (Bush et al. 2008). Other species, such as *G. triacanthos* and *U. parvifolia* , showed a trend of decrease in growth in response to the local climatic conditions in some cities, while *C. australis* , *J. mimosifolia* , and *P. acerifolia* , for example, showed no significant growth reductions under continuous stress, indicative of these species' adaptation to climatic stress.

Our results showed that cities can expand the realized niche of the species, as it is suggested by modelling studies (e.g., Kim et al. 2020; Lin et al. 2019; Yang 2009; Zhang and Brack 2021). For example, *G. triacanthos* , a widely planted urban tree known for its hardiness and tolerance to drought and salinity (Blair 1990), had its fastest growth in Mildura, the driest and second hottest city in our study. This growth could be explained by the location of the trees within the city; *G. triacanthos* was found in streets with large nature strips in Mildura. *Pyrus calleryana* showed high growth variability across cities, potentially driven by climate differences and tree age. While it is generally observed that young trees exhibit faster growth rates than mature trees due to resource allocation and developmental stage (Hartshorn et al. 1985; O'Brien et al. 1995; Smith et al. 2019), this relationship can be species‐specific and influenced by shade tolerance. For example, shade‐tolerant species may not display accelerated growth in early development compared to more light‐demanding species (Kerstiens 2001). Thus, the observed faster growth rates in young trees of *P. calleryana* —a tolerant of partial shade (Boyce and Ocasio 2020)—should be interpreted cautiously and within the context of its specific growth strategy. In contrast, old trees had slower growth, consistent with reduced vigor and resource limitations (Munné‐Bosch 2018), as well as hydraulic limitation (i.e., the increasing resistance to water transport in taller trees) (Ryan and Yoder 1997; Yoder et al. 1994), which can limit photosynthesis and growth, especially in the upper canopy.

Old urban trees may have experienced stress over time, potentially limiting their growth rates. Alternatively, there might be species adaptations to specific locations that enhance growth. Notably, *R. pseudoacacia* had the fastest average growth rate across all cities despite being the species with the coolest climatic conditions, as inferred from its known global distribution (Esperon‐Rodriguez et al. 2022). While previous research has shown that ring‐porous species can be more vulnerable to drought than diffuse‐porous species (Bush et al. 2008; Michelot et al. 2012; Nitschke et al. 2017), other factors such as leaf traits, rooting depth, or tree size also influence growth and survival under drought conditions (Grote et al. 2016; Lopez‐Iglesias et al. 2014; Padilla and Pugnaire 2007). Nonetheless, although *R. pseudoacacia* is generally considered drought‐sensitive and showed greater variability, it had the fastest growth in Melbourne—the coolest city—suggesting that its performance may be strongly climate‐dependent and optimal under cooler and more humid urban conditions. In contrast, *M. grandiflora* had the slowest growth rates of all 10 species, and similarly to *R. pseudoacacia* , this species had its fastest growth rate in the coolest city (Melbourne). *Magnolia grandiflora* has a slow growth rate (Nassar et al. 2009) compared to other species in this study, which may be related to its evergreen habit and resource‐conservative strategy. Despite this, *M. grandiflora* is widely planted across Australian cities, likely because of aesthetics (Esperon‐Rodriguez et al. 2019).

### 4.2 Drought Response Indices

Our analysis of drought response indices revealed that trees exhibited high resistance and recovery in at least one city where they were planted. The ten species examined showed varying capacities to return to pre‐drought growth levels following drought events. *Pyrus calleryana* demonstrated the highest resilience, followed by *C. australis* and *P. acerifolia* . In contrast, *M. grandiflora* and *R. pseudoacacia* had negative resilience values, indicating difficulty in recovering from drought. Most species exhibited high recovery, except *L. styraciflua* in Mildura—the driest city—while resilience values were generally low to moderate, suggesting limited capacity to fully regain pre‐drought growth rates. These findings highlight interspecific variability in drought responses and reinforce the need for species‐specific strategies in urban forest planning under climate change.

Some species, such as *C. australis* , *J. mimosifolia* , and *P. acerifolia* , showed relatively stable growth across cities with contrasting climates. This may reflect their capacity to thrive under a wide range of local environmental conditions and a high level of adaptation to climatic stress (Roloff 2013). Urban environments can facilitate adaptation through mechanisms such as reduced competition and assisted migration (Booth 2017). However, such adaptability does not necessarily confer higher drought resistance, recovery, or resilience. Tolerance to diverse conditions may come at the cost of reduced performance under specific drought characteristics. Trade‐offs in resource allocation might favor survival over rapid post‐drought recovery (Zlobin 2024), while external factors like competition, pests, diseases, and urban management practices may also influence tree growth (Hilbert et al. 2019; Pretzsch et al. 2015; Raum et al. 2023). These findings illustrate the complexity of drought responses in urban trees and underscore the importance of considering both species‐wide adaptive traits and local environmental contexts when assessing resilience.

We acknowledge that species‐level differences identified here may be influenced by our selection of drought years and the time window used to calculate the SPEI. When drought conditions are prolonged and severe, recovery capacity can be impaired, leading to lasting reductions in growth even after climatic conditions improve (Fini et al. 2009; Marchin et al. 2022). This can impair tree functioning and potentially reduce vigor (Gillner et al. 2013; Michelot et al. 2012). While slower growth may increase vulnerability to secondary stressors such as pests or diseases—and thereby elevate mortality risk—it does not necessarily lead to death.

### 4.3 Tree Growth Model

We found a significant effect of PWM and *I*_P_ on tree growth, highlighting the importance of wet conditions in urban tree growth. However, finding that extreme climate events characterized by hot and dry conditions also had a positive effect on growth seems counterintuitive. This could indicate that trees, particularly temperate‐deciduous trees, in these urban environments are adapted to or benefit from warmer conditions. However, this fast growth could represent a delayed response in growth from milder conditions during the years preceding the extreme climate events. The effect of these extreme conditions on growth also had a persistent impact when conditions were cooler and wetter in southeastern Australia during La Niña. La Niña years showed a significant positive effect on growth, likely due to increased rainfall associated with these events (Huang et al. 2024). However, growth was lower during these years compared to previous warmer and drier years, mainly in the hottest and driest cities. These findings also suggest a delayed response to the previous hot and dry years. Alternatively, urban trees could be decoupled from macroclimates and extreme climate events because urban tree management practices (e.g., irrigation, climate adaptation strategies) can mitigate the impacts of such events (Esperon‐Rodriguez, Sharmin, et al. 2025; Livesley et al. 2021).

### 4.4 Caveats and Limitations

While we have shown different growth responses of 10 tree species to climate in Australian cities, we acknowledge some caveats for our study. First, we highlight that given the limited number of trees available in each city, it was not possible to standardize tree age across all sampled trees. The effect of age on growth underscores the potential importance of maintaining a diverse age structure within urban forests for ensuring ongoing growth and ecosystem services (Mänttäri et al. 2023). We recognize that tree size is often a critical determinant of growth and ecosystem service provision (Franceschi et al. 2022). Therefore, further research is needed to disentangle the relative contributions of age and size to tree growth in these urban environments. Furthermore, we acknowledge that younger trees in our sample may not have experienced sufficient climate variability to show clear growth responses to environmental conditions. Such trees can be influenced by local factors (e.g., shading, soil moisture) rather than broader climate patterns. This limitation was partially mitigated by our minimum diameter threshold and the removal of early growth years, but it remains an important consideration when interpreting our results.

Second, urban environments are quite heterogeneous, and environmental conditions can vary greatly within cities; thus, the location of trees can drive differential species responses. For example, the high mortality of *Ailanthus altissima* in Baltimore, Maryland (US), was explained by its occurrence along highways (Nowak et al. 2004). By standardizing the planting context—i.e., sampling only street trees—we aimed to minimize this issue; however, including trees in parks could provide evidence on how trees respond when planted in areas with high soil volume and more access to water. Third, additional environmental factors that were not accounted for here may limit species growth, including pollution and light availability, which influence tree growth (Moser et al. 2016). Light availability could explain some of our results. In cities like Mildura, with fewer buildings and more open spaces, trees receive more direct sunlight throughout the day. Greater light availability can lead to faster growth rates (Cronin and Lodge 2003). More light also allows for increased photosynthetic activity, potentially leading to better overall tree health and growth (Kirschbaum 2010).

Fourth, management actions (e.g., irrigation) in cities can mitigate the impacts of extreme climate events (Gao and Santamouris 2019). If water is provided through active management actions or other sources, including water provision from urban residents, such as reported in Penrith and Sydney (Esperon‐Rodriguez, Sharmin, et al. 2025), species can survive in dry cities. Unfortunately, specific data on irrigation were not available for this study; therefore, we cannot quantify the potential influence of supplemental watering, either from formal irrigation systems or informal sources such as residents, on tree growth. Alternatively, deep rooting may provide water access for urban trees during dry periods (Day et al. 2009). Fifth, although information on provenances was not available for the species in this study, we acknowledge that provenances show different levels of adaptability to climates markedly different from those of their origin (Ahrens et al. 2019). A widely distributed tree species may be chosen by practitioners based on the idea of its robustness, yet the origin of the genetic material used for planting may not reflect that tolerance. Sixth, the sensitivity of the drought response indices used here can vary depending on the severity of the drought events (Trotsiuk et al. 2018); however, these variations require further investigation in urban environments. Finally, functional traits that were not accounted for here can also be related to the species' performance in cities by increasing their climatic tolerance and enhancing growth (Marchin et al. 2022; Simovic et al. 2024).

Despite these caveats, our findings highlight the complex interactions between climatic, environmental, and anthropogenic factors in shaping urban tree growth. City‐specific factors (e.g., management practices, urban design, local climate) can play a significant role in tree growth, highlighting the importance of considering local conditions in urban forestry. Our results underscore the importance of tailored urban forestry strategies, such as diverse species selection, to enhance the resilience and health of urban trees in the face of climate change and other stressors, including different extreme climate events. The different responses of the studied urban tree species to climatic conditions and extreme climate events have implications for their ability to provide ecosystem services today and in the future. Urban forest managers must face the challenge of managing current tree species to reduce the effects of climate change, drought, and heat, and select different combinations of species that can withstand rising temperatures and drought while still providing benefits to urban residents, like heat mitigation.

### 4.5 Practical Recommendations

Our results suggest that a wide range of exotic tree species can thrive in Australian urban environments. Urban planners should prioritize increasing species diversity in urban forests to enhance resilience to climate change, pests, and diseases (Paquette et al. 2021; Sjöman et al. 2016). This can be achieved by incorporating a mix of native and non‐native species that are well‐suited to local climate conditions and soil types (Esperon‐Rodriguez, Gallagher, et al. 2025). To ensure long‐term sustainability within the urban forests, we recommend avoiding monocultures and promoting a diverse age structure by including varied species and growth rates (Thompson et al. 2009). Depending upon planting context, having a mix of fast‐ and slow‐growing tree species can create a multi‐layered or multi‐aged forest structure, enhancing biodiversity and ensuring a continuous provision of ecosystem services over time as different species reach maturity at different rates (Esperon‐Rodriguez, Gallagher, et al. 2025). This approach not only enhances resilience but also overall ecological functionality (Messier et al. 2019) and can be achieved by matching the choice of planting material to sites, a long‐recognized approach within forestry and silviculture (McLeod 2000). Finally, we also emphasize the importance of considering functional traits and ecosystem services during the species selection process (Farrell et al. 2022).

Our findings also suggest that additional factors may influence species' performance and growth in urban environments. Functional traits, such as small leaves and high wood density, can indicate adaptive capacity to local urban conditions (Esperon‐Rodriguez et al. 2020). However, the availability of trait data in urban settings remains limited, with research focusing on a narrow set of species and traits. Future studies should prioritize expanding urban trait databases to better understand species' functional responses to climate stressors and urban management practices. Additionally, urban management strategies, such as irrigation, can mitigate the effects of unfavorable climate conditions and potentially influence plant functional traits, thereby shaping species' persistence and growth in cities. Advancing research on these interactions will be crucial for optimizing tree selection and management in a changing climate.

## 5 Conclusions

Our results reveal long‐term growth patterns of different tree species in urban environments across Australian cities, providing insights into climate resilience and informing urban forest management practices. While identifying species with consistently positive growth responses across a range of climate conditions can inform species selection, our findings emphasize the importance of prioritizing species diversity to enhance urban forest resilience and ecosystem service provision. Urban forest management should prioritize practices that promote the health and vigor of a diverse range of trees. These practices include appropriate irrigation and fertilization strategies, proactive pest and disease management, and canopy management techniques to mitigate the impacts of extreme climate events. Recognizing that ecosystem services are broad, management practices should acknowledge that these extend beyond tree growth and include aesthetic, social, and environmental factors. Emphasizing diverse species selection and appropriate urban forestry management practices will ensure socio‐economic benefits to governments and urban residents.

## Author Contributions

**Manuel Esperon‐Rodriguez:** conceptualization, data curation, formal analysis, funding acquisition, investigation, methodology, project administration, resources, validation, visualization, writing – original draft, writing – review and editing. **Matthew Brookhouse:** conceptualization, formal analysis, funding acquisition, investigation, methodology, resources, software, supervision, validation, writing – review and editing. **Sally A. Power:** conceptualization, funding acquisition, methodology, resources, supervision, writing – review and editing. **Diego Avi:** data curation, formal analysis. **Thomas Baer:** formal analysis, investigation, methodology, validation, writing – review and editing. **Paul D. Rymer:** conceptualization, investigation, writing – review and editing. **Mark G. Tjoelker:** conceptualization, formal analysis, funding acquisition, investigation, methodology, project administration, resources, supervision, validation, visualization, writing – review and editing.

## Conflicts of Interest

The authors declare no conflicts of interest.

## Supporting information


Data S1.

## Data Availability

The data supporting the findings of this study are openly available in Figshare at https://figshare.com/s/1445f6ad49a4d5018a94.
