# Supplementary material for: Urban Tree Growth and Drought Responses Show Evidence of Climate Resilience
Source: Glob Chang Biol. 2025 Jun 9;31(6):e70281. doi: 10.1111/gcb.70281 (PMC12146951; doi:10.1111/gcb.70281)
Supplement: Supplementary file 1 — Data S1. [file GCB-31-e70281-s001.docx]

**Urban tree growth and drought responses show evidence of climate resilience**

Manuel Esperon-Rodriguez^1,2*^, Matthew Brookhouse^3^, Sally A. Power^1^, Diego Avi^3^,

Thomas Baer^4^, Paul D. Rymer^1^, Mark G. Tjoelker^1^

^1^ Hawkesbury Institute for the Environment, Western Sydney University, Locked Bag 1797, Penrith, NSW 2751, Australia

^2^ School of Science, Western Sydney University. Locked Bag 1797, Penrith, NSW 2751, Australia
^3^ Fenner School of Environment and Society, Australian National University, Acton, 2600, Australia

^4^ Forest Research, Forest Resources and Product Development Group, Northern Research Station, Roslin, Midlothian EH25 9SY, United Kingdom

**Supplemental Table S1**. Species pre-identified as vulnerable or resilient to climate for mean annual temperature (MAT) and annual precipitation (AP) in Australian cities based on assessments of bioclimatic modelling of climate risk (Esperon-Rodriguez M et al. 2022b, Esperon‐Rodriguez et al. 2019).

| **City** | **Species** | **MAT** | **AP** |
| --- | --- | --- | --- |
| Adelaide | *Acer negundo* | Resilient | Resilient |
|  | *Celtis australis* | Resilient | Resilient |
|  | *Gleditsia triacanthos* | Resilient | Vulnerable |
|  | *Jacaranda mimosifolia* | Resilient | Vulnerable |
|  | *Platanus acerifolia* | Resilient | Resilient |
|  | *Pyrus calleryana* | Resilient | Vulnerable |
|  | *Robinia pseudoacacia* | Vulnerable | Vulnerable |
|  | *Ulmus parvifolia* | Resilient | Resilient |
| Mandurah | *Acer negundo* | Vulnerable | Vulnerable |
|  | *Celtis australis* | Vulnerable | Vulnerable |
|  | *Gleditsia triacanthos* | Vulnerable | Vulnerable |
|  | *Jacaranda mimosifolia* | Vulnerable | Vulnerable |
|  | *Liquidambar styraciflua* | Vulnerable | Vulnerable |
|  | *Magnolia grandiflora* | Vulnerable | Vulnerable |
|  | *Platanus acerifolia* | Vulnerable | Vulnerable |
|  | *Pyrus calleryana* | Vulnerable | Vulnerable |
|  | *Ulmus parvifolia* | Vulnerable | Vulnerable |
| Melbourne | *Acer negundo* | Vulnerable | Vulnerable |
|  | *Celtis australis* | Vulnerable | Vulnerable |
|  | *Gleditsia triacanthos* | Vulnerable | Vulnerable |
|  | *Jacaranda mimosifolia* | Resilient | Vulnerable |
|  | *Liquidambar styraciflua* | Vulnerable | Vulnerable |
|  | *Magnolia grandiflora* | Resilient | Vulnerable |
|  | *Platanus acerifolia* | Vulnerable | Vulnerable |
|  | *Pyrus calleryana* | Vulnerable | Vulnerable |
|  | *Robinia pseudoacaic* | Resilient | Vulnerable |
|  | *Ulmus parvifolia* | Resilient | Vulnerable |
| Mildura | *Acer negundo* | Vulnerable | Vulnerable |
|  | *Celtis australis* | Vulnerable | Vulnerable |
|  | *Gleditsia triacanthos* | Vulnerable | Vulnerable |
|  | *Jacaranda mimosifolia* | Resilient | Vulnerable |
|  | *Liquidambar styraciflua* | Vulnerable | Vulnerable |
|  | *Magnolia grandiflora* | Vulnerable | Vulnerable |
|  | *Platanus acerifolia* | Vulnerable | Vulnerable |
|  | *Pyrus calleryana* | Vulnerable | Vulnerable |
|  | *Ulmus parvifolia* | Vulnerable | Vulnerable |
| Sydney | *Acer negundo* | Vulnerable | Vulnerable |
|  | *Celtis australis* | Vulnerable | Resilient |
|  | *Gleditsia triacanthos* | Vulnerable | Vulnerable |
|  | *Jacaranda mimosifolia* | Vulnerable | Vulnerable |
|  | *Liquidambar styraciflua* | Vulnerable | Vulnerable |
|  | *Magnolia grandiflora* | Vulnerable | Vulnerable |
|  | *Platanus acerifolia* | Vulnerable | Resilient |
|  | *Pyrus calleryana* | Vulnerable | Vulnerable |
|  | *Robinia pseudoacaic* | Vulnerable | Vulnerable |
|  | *Ulmus parvifolia* | Vulnerable | Vulnerable |

**Supplemental Table S2**. Details of seven meteorological stations used to collect monthly temperature and precipitation. Data from the Australian Bureau of Meteorology (http://www.bom.gov.au/).

| **City** | **Name** | **Number** | **Opened** | **Latitude** | **Longitude** | **Elevation** |
| --- | --- | --- | --- | --- | --- | --- |
| Adelaide | Adelaide Airport | 23034 | 1955 | 34.95° S | 138.52° E | 2 m |
| Mandurah | Karnet | 9111 | 1963 | 32.44° S | 116.08° E | 286 m |
| Melbourne | Melbourne Airport | 86282 | 1970 | 37.67° S | 144.83° E | 113 m |
| Mildura | Mildura Airport | 76031 | 1946 | 34.24° S | 142.09° E | 50 m |
| Parramatta | Parramatta North (Masons Drive) | 66124 | 1965 | 33.79° S | 151.02° E | 55 m |
| Penrith | Prospect Reservoir | 67019 | 1887 | 33.82° S | 150.91° E | 61 m |
| Sydney | Sydney Airport AMO | 66037 | 1929 | 33.95° S | 151.17° E | 6 m |

**Supplemental Table S3**. Type of climate according to the de Martonne Aridity Index (*I*_DM_) (Croitoru et al. 2013).

| **Climate type** | ***I*_DM_ values** |
| --- | --- |
| Arid | *I*_DM_ < 10 |
| Semi-arid | 10  ≤ *I*_DM_ ≤20 |
| Mediterranean | 20  ≤ *I*_DM_ ≤24 |
| Semi-humid | 24  ≤ *I*_DM_ ≤28 |
| Humid | 28  ≤ *I*_DM_ ≤35 |
| Very humid | 35  ≤ *I*_DM_ ≤55 |
| Extremely humid | *I*_DM_ > 55 |

**Supplemental Table S4**. Number of tree rings identified for 10 individual trees (when possible) of ten urban tree species planted in seven Australian cities.

| **Species** | **Tree** | **Adelaide** | **Mandurah** | **Melbourne** | **Mildura** | **Parramatta** | **Penrith** | **Sydney** |
| --- | --- | --- | --- | --- | --- | --- | --- | --- |
| *Acer negundo* | 1 | 20 | 26 |  |  | 37 | 31 |  |
|  | 2 |  | 19 |  |  | 41 | 31 | 17 |
|  | 3 |  | 24 |  |  | 30 | 20 | 15 |
|  | 4 |  | 28 |  |  | 30 | 25 | 19 |
|  | 5 |  |  |  |  | 27 | 27 | 20 |
|  | 6 |  |  |  |  | 11 | 23 | 16 |
|  | 7 |  |  |  |  | 25 | 25 | 21 |
|  | 8 |  |  |  |  | 33 | 21 | 16 |
|  | 9 |  |  |  |  | 21 | 20 | 26 |
|  | 10 |  |  |  |  | 24 | 28 | 16 |
| *Celtis australis* | 1 | 9 |  |  |  | 20 | 35 | 37 |
|  | 2 | 45 |  |  |  | 25 | 25 | 31 |
|  | 3 | 18 |  |  |  | 35 | 26 | 34 |
|  | 4 | 37 |  |  |  | 27 | 26 | 36 |
|  | 5 | 46 |  |  |  | 32 | 36 | 36 |
|  | 6 | 47 |  |  |  | 28 | 36 | 35 |
|  | 7 | 25 |  |  |  | 32 | 36 | 34 |
|  | 8 | 22 |  |  |  | 30 | 36 | 36 |
|  | 9 | 22 |  |  |  | 34 | 36 | 36 |
|  | 10 | 13 |  |  |  | 36 | 18 | 30 |
| *Gleditsia triacanthos* | 1 | 34 | 10 | 25 | 18 | 19 | 31 |  |
|  | 2 | 29 | 28 | 24 | 7 | 20 | 33 |  |
|  | 3 | 18 | 23 | 24 | 6 | 14 | 31 |  |
|  | 4 | 33 | 30 | 23 | 7 | 8 | 31 | 10 |
|  | 5 | 32 | 30 | 22 | 7 | 17 | 30 | 31 |
|  | 6 | 30 | 13 | 23 | 8 |  | 32 | 33 |
|  | 7 | 32 | 31 |  | 7 |  | 31 | 29 |
|  | 8 | 33 | 29 |  |  |  | 30 | 27 |
|  | 9 | 31 | 22 |  |  |  | 26 | 22 |
|  | 10 | 32 | 12 |  |  |  | 35 | 34 |
| *Jacaranda mimosifolia* | 1 | 12 | 15 |  | 26 | 20 | 23 | 28 |
|  | 2 | 16 | 17 |  | 17 | 28 | 21 | 21 |
|  | 3 | 36 | 17 |  | 31 | 45 | 30 | 26 |
|  | 4 | 20 | 18 |  | 38 | 16 | 40 | 33 |
|  | 5 | 14 | 14 | 21 | 35 | 30 | 28 | 36 |
|  | 6 | 10 | 21 | 21 | 36 | 30 | 26 | 28 |
|  | 7 | 32 | 6 | 26 | 22 | 28 | 5 | 24 |
|  | 8 | 13 | 12 | 12 | 11 | 22 | 26 | 33 |
|  | 9 | 39 | 8 | 16 | 18 | 31 | 40 | 28 |
|  | 10 | 25 | 30 | 18 | 20 | 28 | 43 | 22 |
| *Liquidambar styraciflua* | 1 |  | 33 | 29 | 18 | 27 |  | 50 |
|  | 2 |  | 14 | 31 | 39 | 24 | 24 | 39 |
|  | 3 |  | 21 | 26 |  | 26 | 63 | 49 |
|  | 4 |  |  | 26 |  | 38 | 46 | 31 |
|  | 5 |  |  | 18 |  | 32 | 34 | 61 |
|  | 6 |  |  | 17 |  | 32 | 18 | 40 |
|  | 7 |  |  |  |  | 27 | 31 | 27 |
|  | 8 |  |  | 14 |  | 32 | 30 | 39 |
|  | 9 |  |  | 12 |  | 22 | 30 | 41 |
|  | 10 |  |  | 30 |  | 39 |  | 45 |
| *Magnolia grandiflora* | 1 | 53 | 18 | 29 |  | 22 | 56 | 26 |
|  | 2 | 48 | 17 | 28 |  | 35 | 31 | 57 |
|  | 3 | 41 |  | 31 |  | 30 | 48 | 58 |
|  | 4 | 39 |  | 21 |  |  | 48 | 67 |
|  | 5 |  |  | 26 |  | 27 | 35 | 65 |
|  | 6 |  |  | 30 |  | 25 | 28 | 51 |
|  | 7 |  |  | 33 |  | 21 | 46 | 53 |
|  | 8 |  |  | 37 |  | 22 | 43 | 70 |
|  | 9 |  |  | 17 |  | 23 | 57 | 39 |
|  | 10 |  |  | 29 |  | 25 |  | 56 |
| *Platanus acerifolia* | 1 | 13 | 9 | 29 | 23 | 12 | 41 | 14 |
|  | 2 | 13 | 11 | 31 | 10 | 7 | 15 | 27 |
|  | 3 | 12 | 8 | 32 | 9 | 12 | 13 | 26 |
|  | 4 | 17 | 11 |  | 21 | 10 | 14 | 22 |
|  | 5 | 16 | 16 | 30 | 29 | 9 | 10 | 26 |
|  | 6 | 22 | 15 | 32 | 15 | 12 | 10 | 23 |
|  | 7 | 21 | 14 | 42 | 21 | 12 | 10 | 26 |
|  | 8 | 21 |  | 40 | 26 | 13 | 9 | 24 |
|  | 9 | 22 | 30 | 41 | 25 | 18 | 11 | 22 |
|  | 10 | 22 | 30 | 33 | 22 | 16 | 9 | 19 |
| *Pyrus calleryana* | 1 | 21 | 18 | 27 | 11 | 17 | 18 | 9 |
|  | 2 | 25 | 16 | 26 | 8 | 17 | 17 | 9 |
|  | 3 | 22 | 20 | 31 | 11 | 10 | 19 | 6 |
|  | 4 | 24 | 18 | 31 | 8 | 11 | 22 | 7 |
|  | 5 | 29 | 15 | 33 | 12 | 18 | 18 | 9 |
|  | 6 | 30 | 18 | 30 | 8 | 16 | 19 | 9 |
|  | 7 | 37 |  | 26 | 9 | 16 | 22 | 14 |
|  | 8 | 19 |  | 26 | 9 | 16 | 20 |  |
|  | 9 | 41 |  | 34 | 10 | 12 | 16 | 11 |
|  | 10 | 29 |  | 9 | 12 | 16 | 12 | 8 |
| *Robinia pseudoacacia* | 1 | 12 | 21 | 10 |  | 10 | 14 | 16 |
|  | 2 | 34 | 22 | 13 |  | 9 | 22 | 12 |
|  | 3 | 7 | 27 | 21 |  | 16 | 17 | 14 |
|  | 4 | 40 | 26 | 23 |  |  | 10 |  |
|  | 5 | 6 |  | 16 |  | 23 | 23 | 26 |
|  | 6 | 11 |  | 18 |  |  | 17 | 18 |
|  | 7 | 6 |  | 12 |  |  | 17 | 24 |
|  | 8 | 45 |  | 12 |  | 18 | 17 | 17 |
|  | 9 | 29 |  | 20 |  | 9 | 37 | 19 |
|  | 10 | 32 |  | 15 |  | 18 |  | 37 |
|  | 11 |  |  |  |  | 10 |  | 13 |
| *Ulmus parvifolia* | 1 | 13 | 22 | 28 | 10 | 24 | 15 | 45 |
|  | 2 | 38 | 23 | 35 | 11 | 18 | 16 | 46 |
|  | 3 | 39 | 30 | 31 | 9 | 26 | 11 | 47 |
|  | 4 | 42 | 28 | 30 | 19 | 25 | 11 |  |
|  | 5 | 38 |  | 31 | 12 |  | 10 | 46 |
|  | 6 | 33 | 24 | 30 | 12 |  | 11 | 33 |
|  | 7 | 39 | 28 | 34 | 22 | 39 | 12 | 41 |
|  | 8 | 39 | 29 | 33 | 15 | 43 | 11 | 34 |
|  | 9 | 32 | 33 | 31 |  |  | 13 | 46 |
|  | 10 | 40 | 32 | 29 |  |  | 12 | 44 |
|  | 11 |  |  | 26 |  |  |  |  |
| **Average** |  | 27 | 21 | 26 | 17 | 23 | 25 | 30 |
| **Standard Deviation** |  | 12 | 7 | 8 | 9 | 9 | 12 | 15 |

**Supplemental Table S5**. Average annual basal area increment (BAI; cm^2^) and standard deviation (in brackets) of ten urban tree species planted in seven Australian cities. Note that not all species were found in every city.

| **Species** | **Adelaide** | **Mandurah** | **Melbourne** | **Mildura** | **Parramatta** | **Penrith** | **Sydney** |
| --- | --- | --- | --- | --- | --- | --- | --- |
| *Acer negundo* | 0.12 (0.09) | 0.16 (0.12) | - | - | 0.07 (0.1) | 0.07 (0.1) | 0.15 (0.12) |
| *Celtis australis* | 0.07 (0.08) | - | - | - | 0.07 (0.08) | 0.08 (0.09) | 0.09 (0.08) |
| *Gleditsia triacanthos* | 0.11 (0.09) | 0.09 (0.08) | 0.07 (0.04) | 0.22 (0.21) | 0.09 (0.09) | 0.08 (0.06) | 0.08 (0.05) |
| *Jacaranda mimosifolia* | 0.09 (0.09) | 0.15 (0.11) | 0.1 (0.07) | 0.1 (0.08) | 0.14 (0.11) | 0.13 (0.13) | 0.2 (0.21) |
| *Liquidambar styraciflua* | - | 0.15 (0.13) | 0.15 (0.2) | 0.33 (0.31) | 0.09 (0.11) | 0.1 (0.12) | 0.11 (0.08) |
| *Magnolia grandiflora* | 0.03 (0.03) | 0.06 (0.05) | 0.09 (0.1) | - | 0.03 (0.04) | 0.03 (0.04) | 0.05 (0.06) |
| *Platanus acerifolia* | 0.15 (0.13) | 0.16 (0.17) | 0.09 (0.08) | 0.13 (0.14) | 0.23 (0.2) | 0.15 (0.18) | 0.13 (0.15) |
| *Pyrus calleryana* | 0.1 (0.09) | 0.09 (0.07) | 0.11 (0.08) | 0.16 (0.13) | 0.13 (0.12) | 0.13 (0.1) | 0.12 (0.12) |
| *Robinia pseudoacacia* | 0.12 (0.1) | 0.12 (0.11) | 0.23 (0.16) | - | 0.12 (0.12) | 0.09 (0.08) | 0.13 (0.09) |
| *Ulmus parvifolia* | 0.07 (0.05) | 0.08 (0.06) | 0.09 (0.1) | 0.13 (0.13) | 0.07 (0.08) | 0.12 (0.13) | 0.15 (0.12) |

**Supplemental Table S6.** Results of Dunn's test for multiple comparisons of basal area increment (BAI) among seven cities and 10 tree species planted in Australian cities. The table presents *Z*-values and Bonferroni-adjusted p-values for each pairwise comparison. Significant differences (*P* < 0.05) indicate species pairs with significantly different BAI

| **Groups** | **Z** | **P.unadj** | **P.adj** |
| --- | --- | --- | --- |
| **Cities** | | | |
| Adelaide - Mandurah | -7.25 | **<0.001** | **<0.001** |
| Adelaide - Melbourne | -4.81 | **<0.001** | **<0.001** |
| Mandurah - Melbourne | 2.95 | **0.003** | **0.067** |
| Adelaide - Mildura | -7.77 | **<0.001** | **<0.001** |
| Mandurah - Mildura | -1.37 | **0.17** | 1 |
| Melbourne - Mildura | -4.05 | **<0.001** | **0.001** |
| Adelaide - Parramatta | 2.63 | **0.009** | **0.179** |
| Mandurah - Parramatta | 9.47 | **<0.001** | **<0.001** |
| Melbourne - Parramatta | 7.35 | **<0.001** | **<0.001** |
| Mildura - Parramatta | 9.70 | **<0.001** | **<0.001** |
| Adelaide - Penrith | 4.00 | **<0.001** | **0.001** |
| Mandurah - Penrith | 10.85 | **<0.001** | **<0.001** |
| Melbourne - Penrith | 8.89 | **<0.001** | **<0.001** |
| Mildura - Penrith | 10.84 | **<0.001** | **<0.001** |
| Parramatta - Penrith | 1.24 | **0.215** | 1 |
| Adelaide - Sydney | -6.12 | **<0.001** | **<0.001** |
| Mandurah - Sydney | 2.54 | **0.011** | **0.231** |
| Melbourne - Sydney | -0.74 | **0.458** | 1 |
| Mildura - Sydney | 3.73 | **<0.001** | **0.004** |
| Parramatta - Sydney | -8.93 | **<0.001** | **<0.001** |
| Penrith - Sydney | -10.78 | **<0.001** | **<0.001** |
| **Species** | | | |
| *Acer negundo - Celtis australis* | 3.37 | **0.001** | **0.034** |
| *Acer negundo - Gleditsia triacanthos* | -4.40 | **<0.001** | **<0.001** |
| *Celtis australis - Gleditsia triacanthos* | -8.81 | **<0.001** | **<0.001** |
| *Acer negundo - Jacaranda mimosifolia* | -9.46 | **<0.001** | **<0.001** |
| *Celtis australis - Jacaranda mimosifolia* | -14.80 | **<0.001** | **<0.001** |
| *Gleditsia triacanthos - Jacaranda mimosifolia* | -5.66 | **<0.001** | **<0.001** |
| *Acer negundo - Liquidambar styraciflua* | -4.84 | **<0.001** | **<0.001** |
| *Celtis australis - Liquidambar styraciflua* | -9.34 | **<0.001** | **<0.001** |
| *Gleditsia triacanthos - Liquidambar styraciflua* | -0.48 | 0.629 | 1 |
| *Jacaranda mimosifolia - Liquidambar styraciflua* | 5.19 | **<0.001** | **<0.001** |
| *Acer negundo - Magnolia grandiflora* | 12.44 | **<0.001** | **<0.001** |
| *Celtis australis - Magnolia grandiflora* | 10.17 | **<0.001** | **<0.001** |
| *Gleditsia triacanthos - Magnolia grandiflora* | 19.75 | **<0.001** | **<0.001** |
| *Jacaranda mimosifolia - Magnolia grandiflora* | 26.84 | **<0.001** | **<0.001** |
| *Liquidambar styraciflua - Magnolia grandiflora* | 20.40 | **<0.001** | **<0.001** |
| *Acer negundo - Platanus acerifolia* | -8.21 | **<0.001** | **<0.001** |
| *Celtis australis - Platanus acerifolia* | -13.11 | **<0.001** | **<0.001** |
| *Gleditsia triacanthos - Platanus acerifolia* | -4.35 | **<0.001** | **0.001** |
| *Jacaranda mimosifolia - Platanus acerifolia* | 1.12 | 0.263 | 1 |
| *Liquidambar styraciflua - Platanus acerifolia* | -3.89 | **<0.001** | **0.005** |
| *Magnolia grandiflora - Platanus acerifolia* | -24.39 | **<0.001** | **<0.001** |
| *Acer negundo - Pyrus calleryana* | -6.90 | **<0.001** | **<0.001** |
| *Celtis australis - Pyrus calleryana* | -11.45 | **<0.001** | **<0.001** |
| *Gleditsia triacanthos - Pyrus calleryana* | -2.95 | **0.003** | 0.141 |
| *Jacaranda mimosifolia - Pyrus calleryana* | 2.38 | **0.017** | 0.783 |
| *Liquidambar styraciflua - Pyrus calleryana* | -2.50 | **0.012** | 0.551 |
| *Magnolia grandiflora - Pyrus calleryana* | -22.16 | **<0.001** | **<0.001** |
| *Platanus acerifolia - Pyrus calleryana* | 1.25 | 0.212 | 1 |
| *Acer negundo - Robinia pseudoacacia* | -10.29 | **<0.001** | **<0.001** |
| *Celtis australis - Robinia pseudoacacia* | -14.95 | **<0.001** | **<0.001** |
| *Gleditsia triacanthos - Robinia pseudoacacia* | -6.92 | **<0.001** | **<0.001** |
| *Jacaranda mimosifolia - Robinia pseudoacacia* | -2.03 | **0.042** | 1 |
| *Liquidambar styraciflua - Robinia pseudoacacia* | -6.51 | **<0.001** | **<0.001** |
| *Magnolia grandiflora - Robinia pseudoacacia* | -25.21 | **<0.001** | **<0.001** |
| *Platanus acerifolia - Robinia pseudoacacia* | -2.94 | **0.003** | 0.149 |
| *Pyrus calleryana - Robinia pseudoacacia* | -4.00 | **<0.001** | **0.003** |
| *Acer negundo - Ulmus parvifolia* | -4.16 | **<0.001** | **0.001** |
| *Celtis australis - Ulmus parvifolia* | -8.86 | **<0.001** | **<0.001** |
| *Gleditsia triacanthos - Ulmus parvifolia* | 0.54 | 0.586 | 1 |
| *Jacaranda mimosifolia - Ulmus parvifolia* | 6.66 | **<0.001** | **<0.001** |
| *Liquidambar styraciflua - Ulmus parvifolia* | 1.07 | 0.287 | 1 |
| *Magnolia grandiflora - Ulmus parvifolia* | -20.71 | **<0.001** | **<0.001** |
| *Platanus acerifolia - Ulmus parvifolia* | 5.19 | **<0.001** | **<0.001** |
| *Pyrus calleryana - Ulmus parvifolia* | 3.67 | **<0.001** | **0.011** |
| *Robinia pseudoacacia - Ulmus parvifolia* | 7.80 | **<0.001** | **<0.001** |

**Supplemental Table S7.** Results of Tukey's Honestly Significant Difference (HSD) test for multiple comparisons of three indices of drought respond (resistance, recovey, and resilience) for different drought events with extreme precipitation deficit for ten urban tree species planted in seven Australian cities. The 'diff' column shows the mean difference between pairs of resilience or resistance, while 'lwr' and 'upr' represent the lower and upper bounds of the 95% confidence interval, respectively. The '*P* value' column indicates the adjusted *p*-value for each comparison.

| **Groups** | **diff** | **lwr** | **upr** | ***P* value** |
| --- | --- | --- | --- | --- |
| **Resilience** | | | | |
| *Celtis australis-Acer negundo* | -0.42 | -2.96 | 2.11 | 1.00 |
| *Gleditsia triacanthos-Acer negundo* | -1.90 | -4.19 | 0.38 | 0.18 |
| *Jacaranda mimosifolia-Acer negundo* | -0.80 | -3.02 | 1.41 | 0.97 |
| *Liquidambar styraciflua-Acer negundo* | -0.19 | -2.48 | 2.09 | 1.00 |
| *Magnolia grandiflora-Acer negundo* | 0.03 | -2.26 | 2.32 | 1.00 |
| *Platanus acerifolia-Acer negundo* | -0.11 | -2.32 | 2.10 | 1.00 |
| *Pyrus calleryana-Acer negundo* | -1.01 | -3.30 | 1.28 | 0.90 |
| *Robinia pseudoacacia-Acer negundo* | -0.68 | -2.97 | 1.61 | 0.99 |
| *Ulmus parvifolia-Acer negundo* | -0.14 | -2.35 | 2.07 | 1.00 |
| *Gleditsia triacanthos-Celtis australis* | -1.48 | -3.92 | 0.96 | 0.60 |
| *Jacaranda mimosifolia-Celtis australis* | -0.38 | -2.75 | 1.99 | 1.00 |
| *Liquidambar styraciflua-Celtis australis* | 0.23 | -2.21 | 2.67 | 1.00 |
| *Magnolia grandiflora-Celtis australis* | 0.45 | -1.99 | 2.89 | 1.00 |
| *Platanus acerifolia-Celtis australis* | 0.31 | -2.06 | 2.68 | 1.00 |
| *Pyrus calleryana-Celtis australis* | -0.59 | -3.03 | 1.85 | 1.00 |
| *Robinia pseudoacacia-Celtis australis* | -0.26 | -2.70 | 2.18 | 1.00 |
| *Ulmus parvifolia-Celtis australis* | 0.29 | -2.08 | 2.65 | 1.00 |
| *Jacaranda mimosifolia-Gleditsia triacanthos* | 1.10 | -1.00 | 3.20 | 0.77 |
| *Liquidambar styraciflua-Gleditsia triacanthos* | 1.71 | -0.47 | 3.89 | 0.25 |
| *Magnolia grandiflora-Gleditsia triacanthos* | 1.93 | -0.25 | 4.11 | 0.12 |
| *Platanus acerifolia-Gleditsia triacanthos* | 1.79 | -0.31 | 3.89 | 0.16 |
| *Pyrus calleryana-Gleditsia triacanthos* | 0.89 | -1.29 | 3.07 | 0.94 |
| *Robinia pseudoacacia-Gleditsia triacanthos* | 1.22 | -0.96 | 3.40 | 0.70 |
| *Ulmus parvifolia-Gleditsia triacanthos* | 1.77 | -0.34 | 3.87 | 0.17 |
| *Liquidambar styraciflua-Jacaranda mimosifolia* | 0.61 | -1.49 | 2.71 | 0.99 |
| *Magnolia grandiflora-Jacaranda mimosifolia* | 0.83 | -1.27 | 2.93 | 0.95 |
| *Platanus acerifolia-Jacaranda mimosifolia* | 0.69 | -1.33 | 2.71 | 0.98 |
| *Pyrus calleryana-Jacaranda mimosifolia* | -0.21 | -2.31 | 1.89 | 1.00 |
| *Robinia pseudoacacia-Jacaranda mimosifolia* | 0.12 | -1.98 | 2.22 | 1.00 |
| *Ulmus parvifolia-Jacaranda mimosifolia* | 0.67 | -1.35 | 2.69 | 0.98 |
| *Magnolia grandiflora-Liquidambar styraciflua* | 0.22 | -1.96 | 2.40 | 1.00 |
| *Platanus acerifolia-Liquidambar styraciflua* | 0.08 | -2.02 | 2.18 | 1.00 |
| *Pyrus calleryana-Liquidambar styraciflua* | -0.82 | -3.00 | 1.36 | 0.96 |
| *Robinia pseudoacacia-Liquidambar styraciflua* | -0.49 | -2.67 | 1.69 | 1.00 |
| *Ulmus parvifolia-Liquidambar styraciflua* | 0.06 | -2.05 | 2.16 | 1.00 |
| *Platanus acerifolia-Magnolia grandiflora* | -0.14 | -2.24 | 1.96 | 1.00 |
| *Pyrus calleryana-Magnolia grandiflora* | -1.04 | -3.22 | 1.14 | 0.85 |
| *Robinia pseudoacacia-Magnolia grandiflora* | -0.71 | -2.89 | 1.47 | 0.99 |
| *Ulmus parvifolia-Magnolia grandiflora* | -0.17 | -2.27 | 1.94 | 1.00 |
| *Pyrus calleryana-Platanus acerifolia* | -0.90 | -3.00 | 1.20 | 0.92 |
| *Robinia pseudoacacia-Platanus acerifolia* | -0.57 | -2.67 | 1.53 | 1.00 |
| *Ulmus parvifolia-Platanus acerifolia* | -0.03 | -2.05 | 1.99 | 1.00 |
| *Robinia pseudoacacia-Pyrus calleryana* | 0.33 | -1.85 | 2.51 | 1.00 |
| *Ulmus parvifolia-Pyrus calleryana* | 0.87 | -1.23 | 2.97 | 0.93 |
| *Ulmus parvifolia-Robinia pseudoacacia* | 0.54 | -1.56 | 2.64 | 1.00 |
| **Recovery** | | | | |
| *Celtis australis-Acer negundo* | -0.19 | -1.58 | 1.20 | 1.00 |
| *Gleditsia triacanthos-Acer negundo* | -0.37 | -1.63 | 0.89 | 0.99 |
| *Jacaranda mimosifolia-Acer negundo* | -0.40 | -1.61 | 0.82 | 0.99 |
| *Liquidambar styraciflua-Acer negundo* | -0.26 | -1.51 | 1.00 | 1.00 |
| *Magnolia grandiflora-Acer negundo* | -0.47 | -1.72 | 0.79 | 0.96 |
| *Platanus acerifolia-Acer negundo* | -0.17 | -1.39 | 1.04 | 1.00 |
| *Pyrus calleryana-Acer negundo* | 0.34 | -0.92 | 1.60 | 1.00 |
| *Robinia pseudoacacia-Acer negundo* | -0.59 | -1.85 | 0.67 | 0.87 |
| *Ulmus parvifolia-Acer negundo* | -0.09 | -1.31 | 1.13 | 1.00 |
| *Gleditsia triacanthos-Celtis australis* | -0.18 | -1.52 | 1.16 | 1.00 |
| *Jacaranda mimosifolia-Celtis australis* | -0.21 | -1.51 | 1.10 | 1.00 |
| *Liquidambar styraciflua-Celtis australis* | -0.07 | -1.41 | 1.28 | 1.00 |
| *Magnolia grandiflora-Celtis australis* | -0.28 | -1.62 | 1.06 | 1.00 |
| *Platanus acerifolia-Celtis australis* | 0.02 | -1.29 | 1.32 | 1.00 |
| *Pyrus calleryana-Celtis australis* | 0.53 | -0.81 | 1.87 | 0.95 |
| *Robinia pseudoacacia-Celtis australis* | -0.40 | -1.74 | 0.94 | 0.99 |
| *Ulmus parvifolia-Celtis australis* | 0.10 | -1.20 | 1.40 | 1.00 |
| *Jacaranda mimosifolia-Gleditsia triacanthos* | -0.02 | -1.18 | 1.13 | 1.00 |
| *Liquidambar styraciflua-Gleditsia triacanthos* | 0.12 | -1.08 | 1.32 | 1.00 |
| *Magnolia grandiflora-Gleditsia triacanthos* | -0.10 | -1.29 | 1.10 | 1.00 |
| *Platanus acerifolia-Gleditsia triacanthos* | 0.20 | -0.96 | 1.35 | 1.00 |
| *Pyrus calleryana-Gleditsia triacanthos* | 0.71 | -0.49 | 1.91 | 0.63 |
| *Robinia pseudoacacia-Gleditsia triacanthos* | -0.22 | -1.42 | 0.98 | 1.00 |
| *Ulmus parvifolia-Gleditsia triacanthos* | 0.28 | -0.87 | 1.44 | 1.00 |
| *Liquidambar styraciflua-Jacaranda mimosifolia* | 0.14 | -1.02 | 1.30 | 1.00 |
| *Magnolia grandiflora-Jacaranda mimosifolia* | -0.07 | -1.23 | 1.08 | 1.00 |
| *Platanus acerifolia-Jacaranda mimosifolia* | 0.22 | -0.89 | 1.33 | 1.00 |
| *Pyrus calleryana-Jacaranda mimosifolia* | 0.73 | -0.42 | 1.89 | 0.53 |
| *Robinia pseudoacacia-Jacaranda mimosifolia* | -0.19 | -1.35 | 0.96 | 1.00 |
| *Ulmus parvifolia-Jacaranda mimosifolia* | 0.31 | -0.81 | 1.41 | 1.00 |
| *Magnolia grandiflora-Liquidambar styraciflua* | -0.21 | -1.41 | 0.99 | 1.00 |
| *Platanus acerifolia-Liquidambar styraciflua* | 0.08 | -1.07 | 1.24 | 1.00 |
| *Pyrus calleryana-Liquidambar styraciflua* | 0.59 | -0.61 | 1.79 | 0.82 |
| *Robinia pseudoacacia-Liquidambar styraciflua* | -0.33 | -1.53 | 0.87 | 1.00 |
| *Ulmus parvifolia-Liquidambar styraciflua* | 0.17 | -0.99 | 1.32 | 1.00 |
| *Platanus acerifolia-Magnolia grandiflora* | 0.29 | -0.86 | 1.45 | 1.00 |
| *Pyrus calleryana-Magnolia grandiflora* | 0.81 | -0.39 | 2.01 | 0.45 |
| *Robinia pseudoacacia-Magnolia grandiflora* | -0.12 | -1.32 | 1.08 | 1.00 |
| *Ulmus parvifolia-Magnolia grandiflora* | 0.38 | -0.78 | 1.53 | 0.98 |
| *Pyrus calleryana-Platanus acerifolia* | 0.51 | -0.64 | 1.67 | 0.90 |
| *Robinia pseudoacacia-Platanus acerifolia* | -0.41 | -1.57 | 0.74 | 0.97 |
| *Ulmus parvifolia-Platanus acerifolia* | 0.08 | -1.03 | 1.19 | 1.00 |
| *Robinia pseudoacacia-Pyrus calleryana* | -0.93 | -2.13 | 0.27 | 0.26 |
| *Ulmus parvifolia-Pyrus calleryana* | -0.43 | -1.58 | 0.73 | 0.96 |
| *Ulmus parvifolia-Robinia pseudoacacia* | 0.50 | -0.66 | 1.65 | 0.91 |
| **Resistance** | | | | |
| *Celtis australis-Acer negundo* | 2.44 | -0.97 | 5.85 | 0.36 |
| *Gleditsia triacanthos-Acer negundo* | 1.57 | -1.51 | 4.65 | 0.80 |
| *Jacaranda mimosifolia-Acer negundo* | 1.67 | -1.31 | 4.64 | 0.70 |
| *Liquidambar styraciflua-Acer negundo* | 1.13 | -1.95 | 4.21 | 0.97 |
| *Magnolia grandiflora-Acer negundo* | 2.91 | -0.17 | 5.99 | 0.08 |
| *Platanus acerifolia-Acer negundo* | 2.15 | -0.83 | 5.13 | 0.35 |
| *Pyrus calleryana-Acer negundo* | 1.29 | -1.79 | 4.37 | 0.93 |
| *Robinia pseudoacacia-Acer negundo* | 1.92 | -1.16 | 4.99 | 0.56 |
| *Ulmus parvifolia-Acer negundo* | 2.29 | -0.69 | 5.26 | 0.27 |
| *Gleditsia triacanthos-Celtis australis* | -0.87 | -4.15 | 2.41 | 1.00 |
| *Jacaranda mimosifolia-Celtis australis* | -0.78 | -3.96 | 2.41 | 1.00 |
| *Liquidambar styraciflua-Celtis australis* | -1.32 | -4.60 | 1.97 | 0.94 |
| *Magnolia grandiflora-Celtis australis* | 0.47 | -2.81 | 3.75 | 1.00 |
| *Platanus acerifolia-Celtis australis* | -0.29 | -3.48 | 2.89 | 1.00 |
| *Pyrus calleryana-Celtis australis* | -1.16 | -4.44 | 2.12 | 0.97 |
| *Robinia pseudoacacia-Celtis australis* | -0.53 | -3.81 | 2.75 | 1.00 |
| *Ulmus parvifolia-Celtis australis* | -0.16 | -3.35 | 3.03 | 1.00 |
| *Jacaranda mimosifolia-Gleditsia triacanthos* | 0.10 | -2.73 | 2.93 | 1.00 |
| *Liquidambar styraciflua-Gleditsia triacanthos* | -0.44 | -3.38 | 2.49 | 1.00 |
| *Magnolia grandiflora-Gleditsia triacanthos* | 1.34 | -1.59 | 4.28 | 0.88 |
| *Platanus acerifolia-Gleditsia triacanthos* | 0.58 | -2.25 | 3.41 | 1.00 |
| *Pyrus calleryana-Gleditsia triacanthos* | -0.29 | -3.22 | 2.65 | 1.00 |
| *Robinia pseudoacacia-Gleditsia triacanthos* | 0.35 | -2.59 | 3.28 | 1.00 |
| *Ulmus parvifolia-Gleditsia triacanthos* | 0.71 | -2.11 | 3.54 | 1.00 |
| *Liquidambar styraciflua-Jacaranda mimosifolia* | -0.54 | -3.37 | 2.29 | 1.00 |
| *Magnolia grandiflora-Jacaranda mimosifolia* | 1.25 | -1.58 | 4.07 | 0.90 |
| *Platanus acerifolia-Jacaranda mimosifolia* | 0.48 | -2.24 | 3.20 | 1.00 |
| *Pyrus calleryana-Jacaranda mimosifolia* | -0.38 | -3.21 | 2.45 | 1.00 |
| *Robinia pseudoacacia-Jacaranda mimosifolia* | 0.25 | -2.58 | 3.08 | 1.00 |
| *Ulmus parvifolia-Jacaranda mimosifolia* | 0.62 | -2.10 | 3.34 | 1.00 |
| *Magnolia grandiflora-Liquidambar styraciflua* | 1.78 | -1.15 | 4.72 | 0.60 |
| *Platanus acerifolia-Liquidambar styraciflua* | 1.02 | -1.81 | 3.85 | 0.97 |
| *Pyrus calleryana-Liquidambar styraciflua* | 0.16 | -2.78 | 3.09 | 1.00 |
| *Robinia pseudoacacia-Liquidambar styraciflua* | 0.79 | -2.15 | 3.72 | 1.00 |
| *Ulmus parvifolia-Liquidambar styraciflua* | 1.16 | -1.67 | 3.99 | 0.94 |
| *Platanus acerifolia-Magnolia grandiflora* | -0.76 | -3.59 | 2.07 | 1.00 |
| *Pyrus calleryana-Magnolia grandiflora* | -1.63 | -4.56 | 1.31 | 0.71 |
| *Robinia pseudoacacia-Magnolia grandiflora* | -1.00 | -3.93 | 1.94 | 0.98 |
| *Ulmus parvifolia-Magnolia grandiflora* | -0.63 | -3.46 | 2.20 | 1.00 |
| *Pyrus calleryana-Platanus acerifolia* | -0.86 | -3.69 | 1.97 | 0.99 |
| *Robinia pseudoacacia-Platanus acerifolia* | -0.23 | -3.06 | 2.59 | 1.00 |
| *Ulmus parvifolia-Platanus acerifolia* | 0.14 | -2.58 | 2.85 | 1.00 |
| *Robinia pseudoacacia-Pyrus calleryana* | 0.63 | -2.31 | 3.57 | 1.00 |
| *Ulmus parvifolia-Pyrus calleryana* | 1.00 | -1.83 | 3.83 | 0.97 |
| *Ulmus parvifolia-Robinia pseudoacacia* | 0.37 | -2.46 | 3.20 | 1.00 |

**Supplemental Table S8**. Average values of three indices of drought respond (resistance, recovery, and resilience) for different drought events with extreme precipitation deficit for ten urban tree species planted in seven Australian cities. Note that not all species were found in every city. Drought response indices are unitless.

| **Species** | **City** | **Resistance** | **Recovery** | **Resilience** |
| --- | --- | --- | --- | --- |
| *Acer negundo* | Adelaide | 1.0 | 1.0 | 0.1 |
|  | Mandurah | 1.6 | 2.2 | 0.2 |
|  | Parramatta | 0.9 | 1.0 | 0.6 |
|  | Penrith | 1.8 | 1.8 | 0.4 |
|  | Sydney | 1.1 | 1.2 | 0.3 |
| *Celtis australis* | Adelaide | 1.1 | 1.6 | 1.0 |
|  | Parramatta | 0.8 | 0.9 | 0.0 |
|  | Penrith | 1.0 | 1.3 | 0.3 |
|  | Sydney | 0.9 | 1.6 | 0.2 |
| *Gleditsia triacanthos* | Adelaide | 0.8 | 1.7 | 0.2 |
|  | Mandurah | 0.9 | 1.1 | 0.2 |
|  | Melbourne | 1.1 | 1.4 | -0.1 |
|  | Parramatta | 0.7 | 2.0 | 0.1 |
|  | Penrith | 1.2 | 0.8 | 0.4 |
|  | Sydney | 1.6 | 1.0 | 0.0 |
| *Jacaranda mimosifolia* | Adelaide | 1.2 | 2.8 | 0.5 |
|  | Mandurah | 1.4 | 1.2 | 0.0 |
|  | Melbourne | 1.5 | 1.3 | -0.3 |
|  | Mildura | 1.3 | 1.0 | -0.4 |
|  | Parramatta | 1.1 | 1.2 | -0.1 |
|  | Penrith | 0.6 | 1.5 | 0.4 |
|  | Sydney | 0.8 | 1.0 | 0.1 |
| *Liquidambar styraciflua* | Mandurah | 1.7 | 1.0 | 0.2 |
|  | Melbourne | 1.4 | 1.3 | 0.1 |
|  | Mildura | 0.3 | 0.8 | 0.0 |
|  | Parramatta | 1.0 | 0.7 | 0.3 |
|  | Penrith | 1.2 | 0.9 | 0.1 |
|  | Sydney | 1.3 | 0.3 | 0.4 |
| *Magnolia grandiflora* | Adelaide | 1.4 | 0.9 | -0.2 |
|  | Mandurah | 0.5 | 2.7 | 0.3 |
|  | Melbourne | 0.8 | 1.5 | 0.2 |
|  | Parramatta | 1.9 | 0.9 | -0.7 |
|  | Penrith | 1.3 | 0.7 | 0.0 |
|  | Sydney | 1.1 | 1.1 | 0.0 |
| *Platanus acerifolia* | Adelaide | 0.8 | 1.2 | 0.8 |
|  | Mandurah | 0.9 | 1.2 | 0.1 |
|  | Melbourne | 0.8 | 2.2 | 0.1 |
|  | Mildura | 1.2 | 1.1 | -0.4 |
|  | Parramatta | 1.6 | 0.7 | 0.5 |
|  | Penrith | 0.9 | 1.1 | 0.9 |
|  | Sydney | 1.0 | 2.1 | 0.4 |
| *Pyrus calleryana* | Adelaide | 0.7 | 1.3 | 0.2 |
|  | Mandurah | 2.3 | 1.6 | 0.5 |
|  | Melbourne | 1.2 | 1.0 | 0.2 |
|  | Parramatta | 1.1 | 1.2 | 0.0 |
|  | Penrith | 0.7 | 1.3 | 0.0 |
|  | Sydney | 2.7 | 1.7 | 1.8 |
| *Robinia pseudoacacia* | Adelaide | 0.9 | 0.8 | 0.5 |
|  | Mandurah | 1.3 | 0.9 | -0.1 |
|  | Melbourne | 0.9 | 1.7 | 0.0 |
|  | Parramatta | 0.9 | 1.4 | -0.2 |
|  | Penrith | 1.0 | 1.2 | -0.2 |
|  | Sydney | 1.0 | 1.7 | -0.1 |
| *Ulmus parvifolia* | Adelaide | 1.0 | 0.9 | 0.6 |
|  | Mandurah | 1.0 | 1.7 | 0.4 |
|  | Melbourne | 0.8 | 1.1 | -0.2 |
|  | Mildura | 2.0 | 1.4 | 0.7 |
|  | Parramatta | 0.9 | 4.5 | 0.2 |
|  | Penrith | 1.6 | 0.9 | 0.1 |
|  | Sydney | 1.2 | 1.3 | 0.1 |

**Supplemental Figure S1**. Spatial representation of (**A**) mean interseries correlation (r-bar) and (**B**) Expressed Population Signal (EPS) for each city × species combination. These metrics were used to assess the strength of the common growth signal for each group. Colour intensity represents the magnitude of r-bar and EPS, with a gradient from white (low values) to blue (high r-bar) and orange (high EPS). Gray tiles indicate combinations for which data were not available.

**Supplemental Figure S2**. Distribution of (**A**) mean interseries correlation (r-bar) and (**B**) Expressed Population Signal (EPS) values across all city × species combinations used in the analysis. The histograms illustrate the range and frequency of r-bar and EPS values, with vertical dashed red lines indicating the mean value for each metric. These metrics were used to assess the strength of the common growth signal within each city × species combination.

**Supplemental Figure S3**. Spaghetti plot showing the inter-individual variability in tree-ring width (TRW) chronologies across seven Australian cities and ten urban tree species. Each line represents the annual TRW of a single tree. Plots are faceted by city and species, with the *y*-axis scales allowed to vary freely to accommodate differences in TRW magnitudes. This plot provides a visual representation of the raw tree-ring data and highlights the range of growth patterns within and between groups. Species abbreviations are as follows: AcNe= *Acer negundo*; CeAu= *Celtis australis*; GlTr= *Gleditsia triacanthos*; JaMi= *Jacaranda mimosifolia*; LiSt= Liquidambar styraciflua; MaGr= *Magnolia grandiflora*; PlAc= *Platanus acerifolia*; PyCa= *Pyrus calleryana*; RoPs= *Robinia pseudoacia*; and UlPa= *Ulmus parvifolia*.

**Supplemental Figure S4**. Standardised Precipitation Evapotranspiration Index (SPEI) for two time scales (2- and 3-months) showing the mean, minimum (min), and variance of the mean (var) through time in the city of Adelaide, South Australia. The shaded areas represent prolonged drought periods with consistently low SPEI values across multiple years based on minimum SPEI, mean SPEI, and variance of SPEI. These events were selected to evaluate tree performance under both prolonged and extreme drought conditions


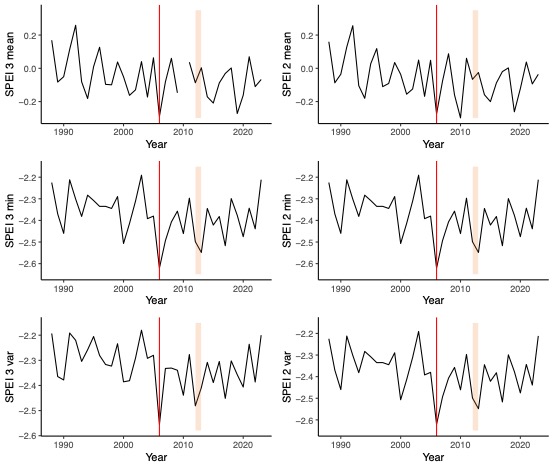


**Supplemental Figure S5**. Standardised Precipitation Evapotranspiration Index (SPEI) for two time scales (2- and 3-months) showing the mean, minimum (min), and variance of the mean (var) through time in the city of Mandurah, Western Australia. The shaded areas represent prolonged drought periods with consistently low SPEI values across multiple years, while the red lines indicate discrete years identified as extreme drought events based on minimum SPEI, mean SPEI, and variance of SPEI. These events were selected to evaluate tree performance under both prolonged and extreme drought conditions


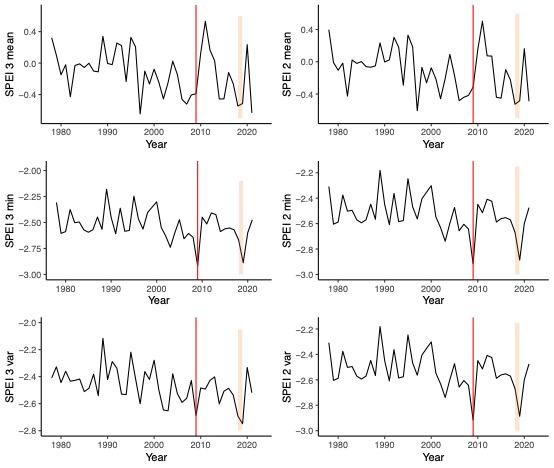


**Supplemental Figure S6**. Standardised Precipitation Evapotranspiration Index (SPEI) for two time scales (2- and 3-months) showing the mean, minimum (min), and variance of the mean (var) through time in the city of Melbourne, Victoria, Australia. The shaded areas represent prolonged drought periods with consistently low SPEI values across multiple years, while the red lines indicate discrete years identified as extreme drought events based on minimum SPEI, mean SPEI, and variance of SPEI. These events were selected to evaluate tree performance under both prolonged and extreme drought conditions

**Supplemental Figure S7**. Standardised Precipitation Evapotranspiration Index (SPEI) for two time scales (2- and 3-months) showing the mean, minimum (min), and variance of the mean (var) through time in the city of Mildura. Victoria. Red lines indicate discrete years identified as extreme drought events based on minimum SPEI, mean SPEI, and variance of SPEI. These events were selected to evaluate tree performance under both prolonged and extreme drought conditions


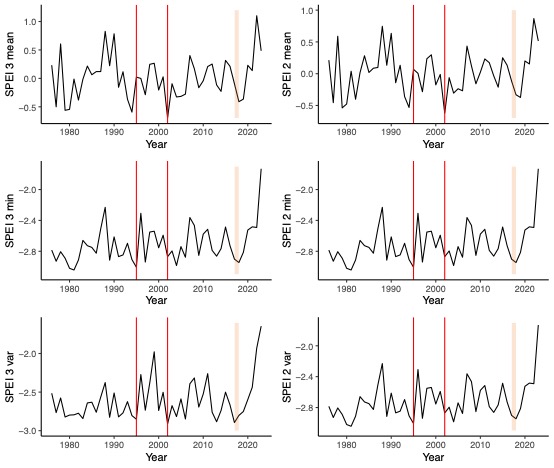


**Supplemental Figure S8.** Standardised Precipitation Evapotranspiration Index (SPEI) for two time scales (2- and 3-months) showing the mean, minimum (min), and variance of the mean (var) through time in the city of Parramatta, New South Wales. The shaded areas represent prolonged drought periods with consistently low SPEI values across multiple years, while the red lines indicate discrete years identified as extreme drought events based on minimum SPEI, mean SPEI, and variance of SPEI. These events were selected to evaluate tree performance under both prolonged and extreme drought conditions

**Supplemental Figure S9.** Standardised Precipitation Evapotranspiration Index (SPEI) for two time scales (2- and 3-months) showing the mean, minimum (min), and variance of the mean (var) through time in the city of Penrith, New South Wales. Red lines indicate discrete years identified as extreme drought events based on minimum SPEI, mean SPEI, and variance of SPEI. These events were selected to evaluate tree performance under both prolonged and extreme drought conditions


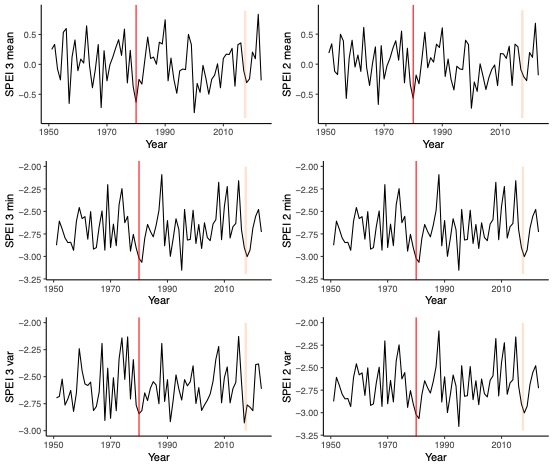


**Supplemental Figure S10.** Standardised Precipitation Evapotranspiration Index (SPEI) for two time scales (2- and 3-months) showing the mean, minimum (min), and variance of the mean (var) through time in the city of Sydney, New South Wales. The shaded areas represent prolonged drought periods with consistently low SPEI values across multiple years, while the red lines indicate discrete years identified as extreme drought events based on minimum SPEI, mean SPEI, and variance of SPEI. These events were selected to evaluate tree performance under both prolonged and extreme drought conditions

**Supplemental Figure S11.** Principal Component Analyses of 10 climatic variables. Values in parentheses indicate variance accounted for by each axis. Annual precipitation = AP, precipitation of the driest month = PDM, precipitation of the driest quarter = PDQ, summer precipitation = SP, precipitation of the wettest month = PWM, mean annual temperature = MAT, maximum temperature of the warmest month = MTWM, minimum temperature of the coldest month = MTCM, Martonne Aridity Index = IDM, and Pinna Combinative Index = IP.

**Supplemental Figure S12**. Differences in detrended annual basal area increment (BAI) of ten urban tree species planted in seven Australian cities*.* Ridgeline plots show the distribution of BAI in each city, with the height and width of the ridgeline corresponding to the density of observations at that particular BAI value; black lines represent the BAI 50^th^ percentile. BAI represents individual tree rings aggregated across all sampled trees of each species in each city. Cities are ordered from low to high annual precipitation. Note that not all species were found in the seven cities. Detrended BAI is unitless. For differences between cities and species based on the Dunn's test, see **Supplemental** **Table S6**.

**Supplemental Figure S13**. Differences of detrended annual basal area increment (BAI) among seven Australian cities (top) and ten urban tree species (bottom). Boxplot shows data distribution with mean values and outliers, while violin plots depict the density of BAI. Cities are ordered from low (Mildura) to high (Sydney) annual precipitation. Species abbreviations are AcNe = *Acer negundo*, CeAu = *Celtis australis*, GlTr = *Gleditsia triacanthos*, JaMi = *Jacaranda mimosifolia*, LiSt = *Liquidambar styraciflua*, MaGr = *Magnolia grandiflora*, PlAc = *Platanus acerifolia*, PyCa = *Pyrus calleryana*, RoPs = *Robinia pseudoacacia*, and UlPa = *Ulmus parvifolia*. Detrended BAI is unitless. For differences between cities and species based on the Dunn's test, see **Supplemental** **Table S6**.
